# Author Correction: Evolutionarily related host and microbial pathways regulate fat desaturation in *C. elegans*

**DOI:** 10.1038/s41467-024-50246-8

**Published:** 2024-07-19

**Authors:** Bennett W. Fox, Maximilian J. Helf, Russell N. Burkhardt, Alexander B. Artyukhin, Brian J. Curtis, Diana Fajardo Palomino, Allen F. Schroeder, Amaresh Chaturbedi, Arnaud Tauffenberger, Chester J. J. Wrobel, Ying K. Zhang, Siu Sylvia Lee, Frank C. Schroeder

**Affiliations:** 1 Boyce Thompson Institute and Department of Chemistry and Chemical Biology, Cornell University, Ithaca, NY 14853 USA; 2 Chemistry Department, College of Environmental Science and Forestry, State University of New York, Syracuse, NY 13210 USA; 3 Department of Molecular Biology and Genetics, Cornell University, Ithaca, NY 14853 USA

**Keywords:** Metabolomics, Metabolic pathways, Caenorhabditis elegans, Fatty acids

Correction to: *Nature Communications* 10.1038/s41467-024-45782-2, published online 19 February 2024

In this article the funding from National Science Foundation (Award 2042101 to F.C.S.) was omitted. The original article has been corrected.

