# Supplementary material for: Evolutionarily related host and microbial pathways regulate fat desaturation in C. elegans
Source: Nat Commun. 2024 Feb 19;15:1520. doi: 10.1038/s41467-024-45782-2 (PMC10876521; doi:10.1038/s41467-024-45782-2)

Supplementary Information

Evolutionarily related host and microbial pathways regulate fat desaturation in *C. elegans*

Bennett W. Fox[1], Maximilian J. Helf[1,#], Russell N. Burkhardt[1,#], Alexander B. Artyukhin[2], Brian J. Curtis[1], Diana Fajardo Palomino[1], Allen F. Schroeder[1], Amaresh Chaturbedi[3], Arnaud Tauffenberger[1], Chester J.J. Wrobel[1], Ying K. Zhang[1], Siu Sylvia Lee[3], Frank C. Schroeder[1,*]

[1]Boyce Thompson Institute and Department of Chemistry and Chemical Biology, Cornell University, Ithaca, New York 14853, United States

[2]Chemistry Department, College of Environmental Science and Forestry, State University of New York, Syracuse, New York 13210, United States

[3]Department of Molecular Biology and Genetics, Cornell University, Ithaca, New York 14853, United States

[#]These authors contributed equally.

*Correspondence to fs31@cornell.edu

Contents

Supplementary Figures 2

Supplementary Methods 14

Supplementary Tables 21

Supplementary References 26

NMR Spectra of Synthetic Compounds 27

Supplementary Figures

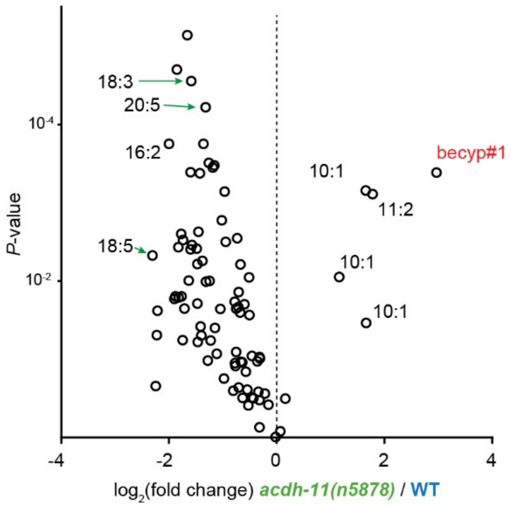

Supplementary Fig. 1. Comparison of free fatty acid profiles of WT and *acdh-11(n5878) C. elegans.*

Volcano plot for 85 fatty acids detected by HPLC-HRMS (negative ion, post-column ion pairing) in the *endo*-metabolomes of WT and *acdh-11(n5878)* mutant *C. elegans* fed BW25113 (WT) *E. coli*. *P*-values were calculated by unpaired, two-sided Welch's t-test; no adjustments were made for multiple comparisons. The most significantly enriched free fatty acid in *acdh-11* mutants is becyp#1; additional cyclopropane-containing medium chain fatty acids are also enriched in *acdh-11* mutants relative to WT *C. elegans*. Several PUFAs were depleted in *acdh-11* relative to WT, e.g., eicosapentaenoic acid (20:5), likely due to reduced growth of these animals when reared on WT *E. coli*. This data is also displayed as part of **Fig. 2C**.

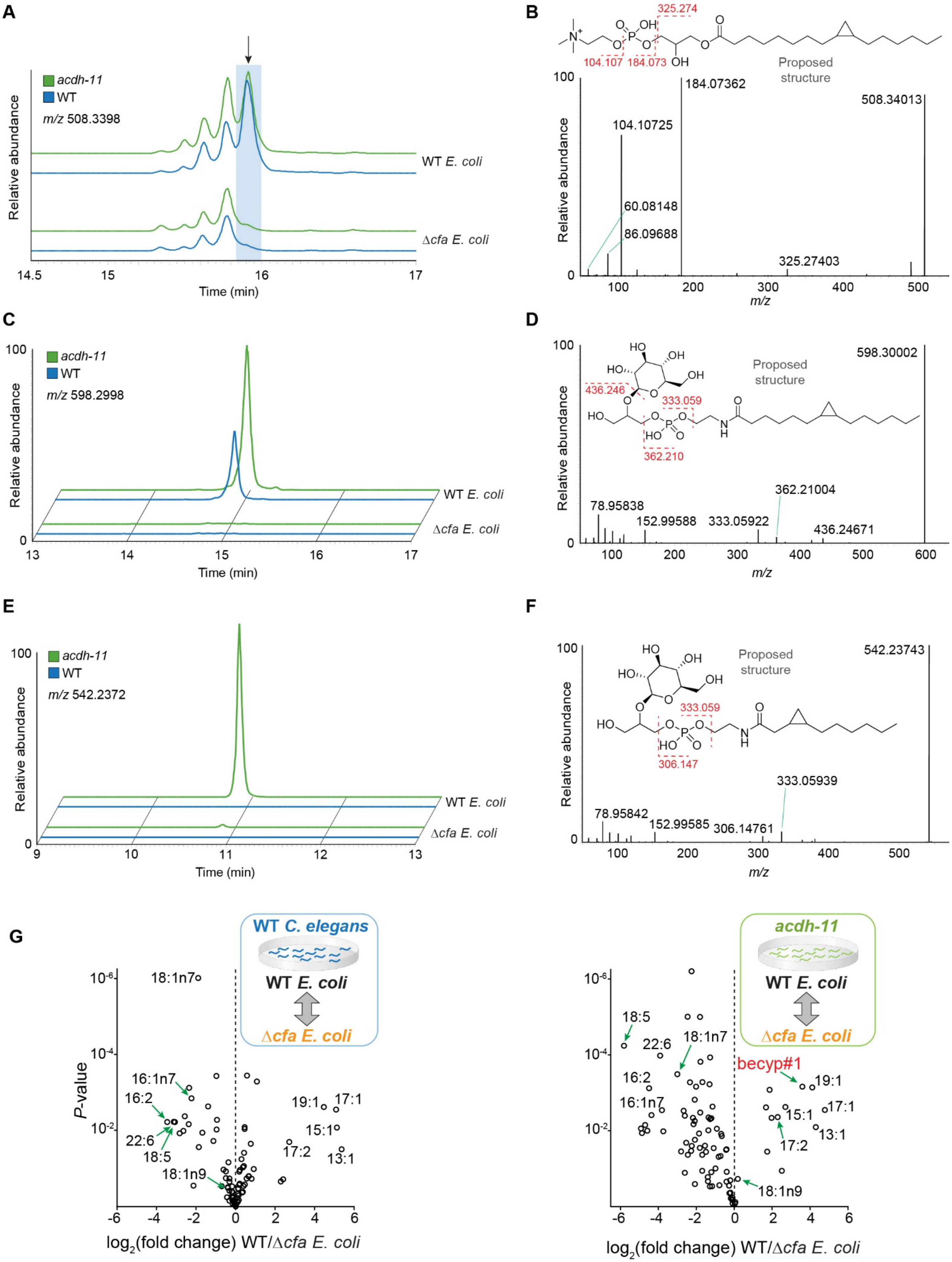
A
acdh-11
WT
m/z 508.3398
Relative abundance
WT E. coli
Δcfa E. coli
Time (min)
B
325.274
104.107
184.073
Proposed structure
184.07362
508.34013
104.10725
60.08148
86.09688
325.27403
Relative abundance
m/z
C
acdh-11
WT
m/z 598.2998
Relative abundance
WT E. coli
Δcfa E. coli
Time (min)
D
436.246
333.059
362.210
Proposed structure
598.30002
362.21004
78.95838
152.99588
333.05922
436.24671
Relative abundance
m/z
E
acdh-11
WT
m/z 542.2372
Relative abundance
WT E. coli
Δcfa E. coli
Time (min)
F
333.059
306.147
Proposed structure
542.23743
333.05939
78.95842
152.99585
306.14761
Relative abundance
m/z
G
WT C. elegans
WT E. coli
Δcfa E. coli
18:1n7
16:1n7
16:2
22:6
18:5
18:1n9
19:1
17:1
15:1
17:2
13:1
P-value
log2(fold change) WT/Δcfa E. coli
acdh-11
WT E. coli
Δcfa E. coli
18:5
22:6
18:1n7
16:2
16:1n7
becyp#1
19:1
17:1
15:1
13:1
17:2
18:1n9
log2(fold change) WT/Δcfa E. coli

Supplementary Fig. 2. Cyclopropane fatty acids are incorporated into host lipids.

A) EICs for *m/z* 508.3398, corresponding to lysophosphatidylcholine (LPC) isomers bearing a singly unsaturated C_{17} acyl group (17:1) with formula $C_{25}H_{51}NO_7P^+$, in extracts of WT and *acdh-11(5878)* mixed-stage cultures reared on WT *E. coli* or *E. coli* Δ*cfa*, as indicated. The feature marked with an arrow is present in animals reared on WT *E. coli* but absent from animals reared on *E. coli* Δ*cfa.*

B) MS/MS spectrum (positive ion mode) of LPC-17:1 with major fragmentation reactions and product ions.

C) EICs for *m/z* 598.2998, corresponding to *N*-acyl glycoglycerophosphoethanolamine (GLEA) bearing a singly unsaturated C_{15} acyl group (15:1) with formula $C_{26}H_{49}NO_{12}P^-$, in extracts of WT and *acdh-11(5878)* mixed-stage cultures reared on WT *E. coli* or *E. coli* Δ*cfa*, as indicated. GLEA-15:1 is abundant in animals reared on WT *E. coli* but absent from animals reared on *E. coli* Δ*cfa.* Structure proposal based on previous characterization of GLEA[47].

D) MS/MS spectrum (negative ion mode) of GLEA-15:1 with major fragmentation reactions and product ions.

E) EICs for *m/z* 542.2372, corresponding to GLEA bearing a singly unsaturated C_{11} acyl group (11:1, becyp#1) with formula $C_{22}H_{41}NO_{12}P^-$, in extracts of WT and *acdh-11(5878)* mixed-stage cultures reared on WT *E. coli* or *E. coli* Δ*cfa*, as indicated. GLEA-becyp#1 is abundant in *acdh-11(n5878)* mutants reared on WT *E. coli*.

F) MS/MS spectrum (negative ion mode) of GLEA-becyp#1 with major fragmentation reactions and product ions.

G) Volcano plots for 85 fatty acids detected by HPLC-HRMS (negative ion, post-column ion pairing) in the *endo*-metabolomes of WT or *acdh-11(n5878)* mutant *C. elegans* fed WT *E. coli* as compared to Δ*cfa E. coli*. *P*-values were calculated by unpaired, two-sided Welch's t-test; no adjustments were made for multiple comparisons. Odd-chain singly unsaturated fatty acids containing the cyclopropane moiety were detected only in animals fed WT *E. coli*, and becyp#1 was enriched only in *acdh-11(n5878)*. Vaccenic (18:1n7) and palmitoleic acid (16:1n7) were increased in animals reared on Δ*cfa E. coli*, consistent with a previous report[48]. In addition, a specific subset of PUFAs was enriched in animals fed Δ*cfa E. coli*, e.g., 18:5 and 22:6, some of which have been previously observed in fatty acid desaturation mutants that accumulate palmitoleic acid[50].

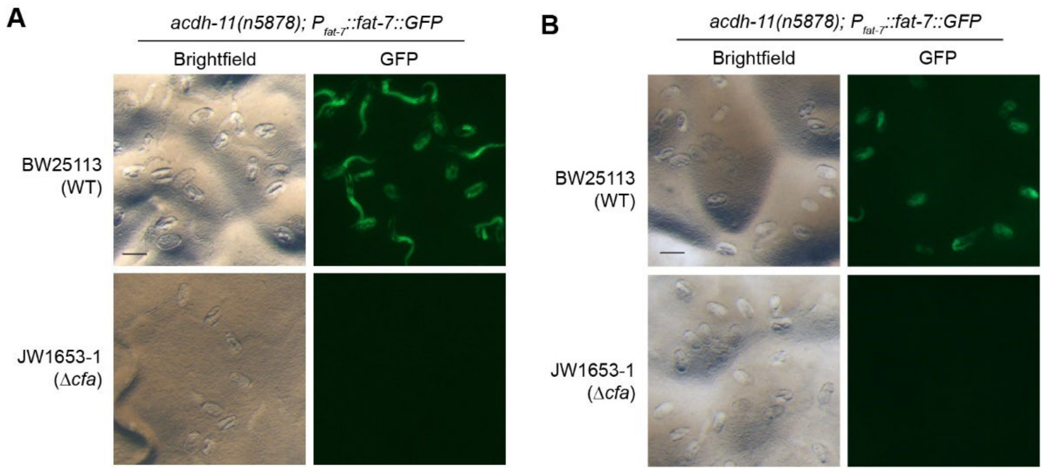

Supplementary Fig. 3. FAT-7::GFP expression in eggs of *acdh-11* mutants is dependent on parental diet.

A) Representative brightfield and fluorescence micrographs of eggs of *acdh-11(n5878);P*$_{fat\text{-}7}$*::fat-7::GFP* mutants reared on BW25113 (WT) or JW1653-1 (Δ*cfa*) *E. coli* for four generations at 20 °C. The scale bar represents 0.05 mm. Six independent experiments were performed.

B) Representative brightfield and fluorescence micrographs of eggs of *acdh-11(n5878);P*$_{fat\text{-}7}$*::fat-7::GFP* mutants reared on BW25113 (WT) or JW1653-1 (Δ*cfa*) *E. coli* for four generations, then switched to the other bacterial diet, maintained at 20 °C. Eggs and larvae had high levels of FAT-7::GFP when the parental generation was reared on WT *E. coli*. The scale bar represents 0.05 mm. Three independent experiments were performed.

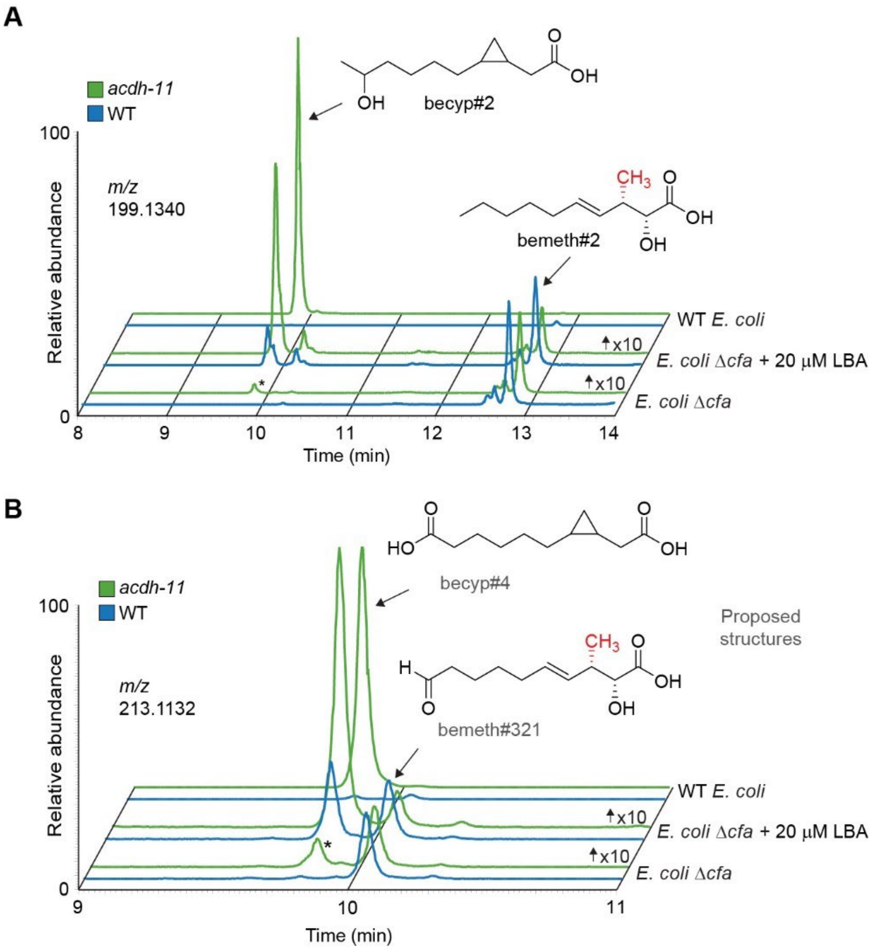

Supplementary Fig. 4. Supplementation with lactobacillic acid (LBA) restores βCPFAs.

A) EICs for *m/z* 199.1340, corresponding to becyp#2, bemeth#2, and structural isomers of $C_{11}H_{19}O_3^-$, in extracts of WT and *acdh-11(n5878)* mixed-stage cultures reared on WT *E. coli, E. coli* Δ*cfa* supplemented with 20 μM LBA (see **Fig. 2**), or *E. coli* Δ*cfa*, as indicated. becyp#2 is strongly enriched in *acdh-11* mutants fed WT *E. coli*, abolished in animals fed *E. coli* Δ*cfa*, and partially restored in animals fed *E. coli* Δ*cfa* supplemented with 20 μM LBA, whereas bemeth#2 is unaffected. An unknown isomer is marked with an asterisk. Y-axes are scaled as indicated to clearly show traces.

B) EICs for *m/z* 213.1123, corresponding to becyp#4, bemeth#321, and structural isomers of $C_{11}H_{17}O_4^-$, in extracts of WT and *acdh-11(n5878)* mixed-stage cultures reared on the same diets as above. Levels of becyp#4 are partially restored by feeding LBA, whereas bemeth#321 is unaffected. An unknown isomer is marked with an asterisk. Y-axes are scaled as indicated to clearly show traces.

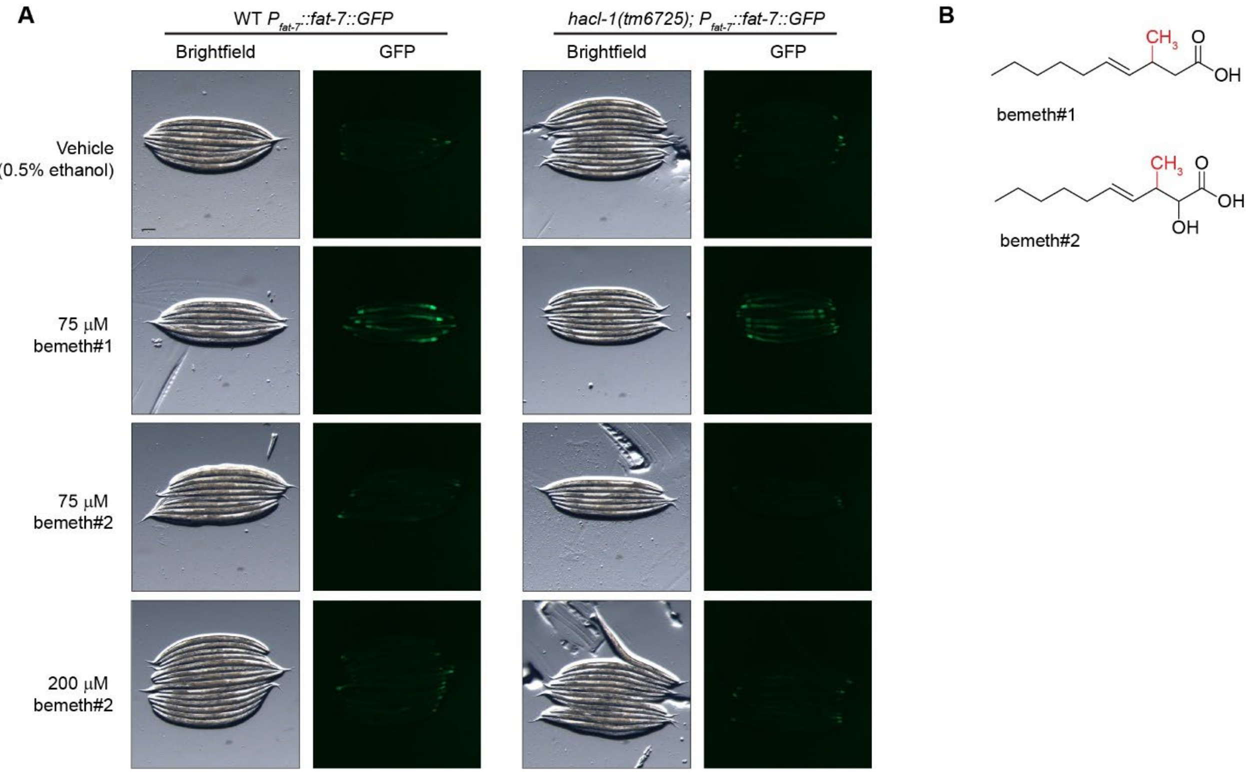

Supplementary Fig. 5. FAT-7::GFP is not induced in *hacl-1* mutants nor following supplementation with bemeth#2.

A) Representative brightfield and GFP fluorescence micrographs of *$P_{fat\text{-}7}$:fat-7::GFP* and *hacl-1(tm6725);$P_{fat\text{-}7}$:fat-7::GFP* adults reared at 25 °C supplemented with vehicle (0.5% ethanol), bemeth#1, or bemeth#2, as indicated. Scale bar represents 0.1 mm. Supplementation with bemeth#2 does not cause overt changes in the abundance of FAT-7::GFP in either genotype. Four independent experiments were performed.

B) Chemical structures of bemeth#1 and its α-hydroxylated derivative, bemeth#2. Several α-hydroxylated βMFAs accumulate in *hacl-1* mutants but do not cause increased *fat-7* expression.

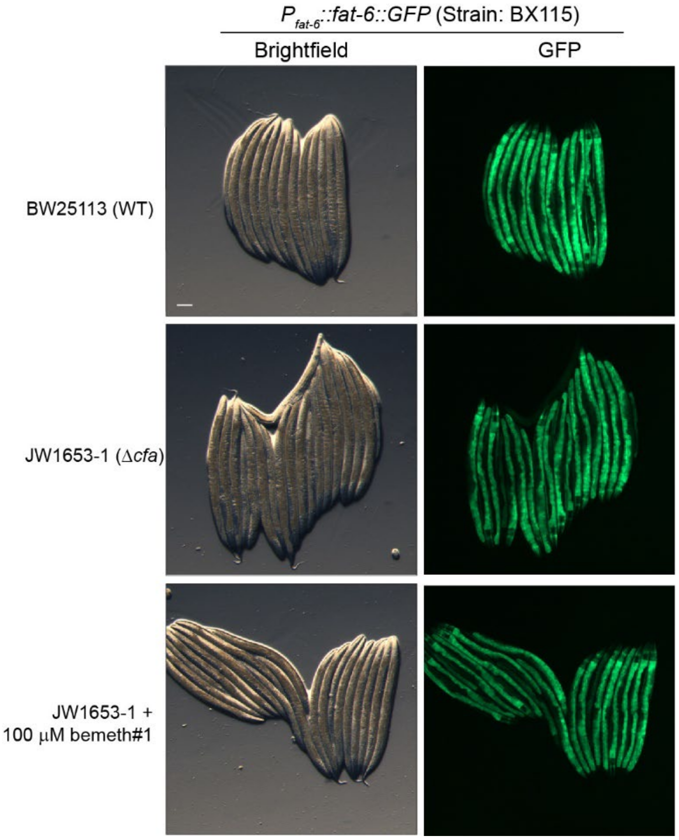

Supplementary Fig. 6. bemeth#1 supplement does not change FAT-6::GFP.

Representative brightfield and fluorescence micrographs of $P_{fat\text{-}6}$::*fat-6::GFP* animals reared on BW25113 (WT), JW1653-1 (Δ*cfa*), or JW1653-1 *E. coli* supplemented with 100 μM bemeth#1. No FAT-6::GFP induction was observed under supplementation conditions. The scale bar represents 0.1 mm. Three independent experiments were performed.

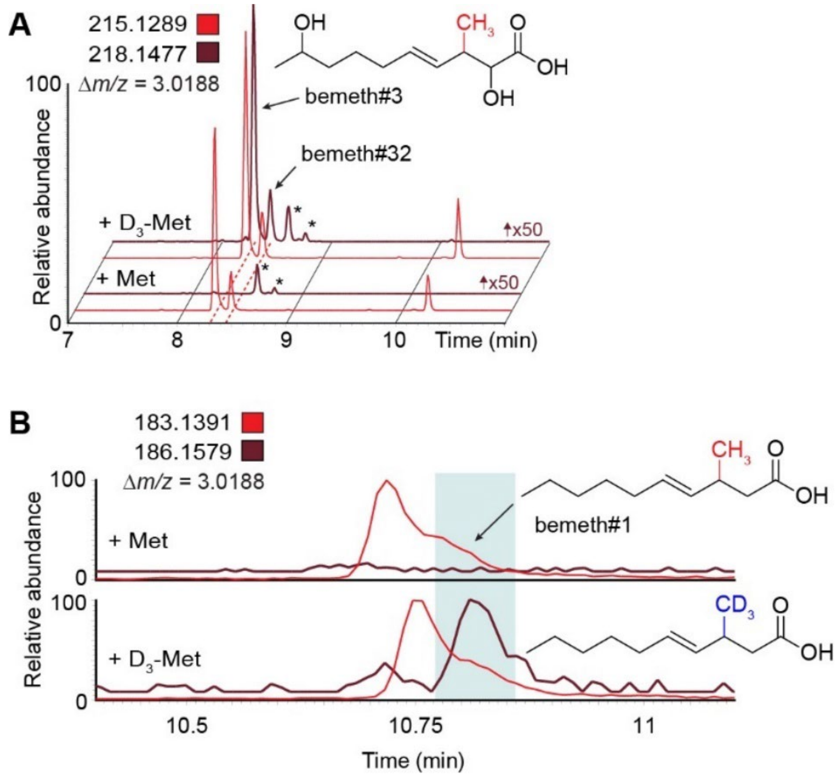

Supplementary Fig. 7. D_3-methyl is incorporated in βMFAs.

A) EICs for *m/z* 215.1289 and 218.1477, corresponding to $C_{11}H_{19}O_4^-$ and D_3-$C_{11}H_{16}O_4^-$, from *exo*-metabolome extracts of *hacl-1(tm6725)* larvae supplemented with methionine (Met) or D_3-methyl-methionine (D_3-Met). Red dashed lines highlight bemeth#3 stereoisomers with D_3-enrichment. EIC Y-axis for *m/z* 218.1477 is scaled 50-fold to clearly show traces for labeled features. Asterisks mark unrelated features present in both Met- and D_3-Met-supplemented samples.

B) EICs for *m/z* 183.1391 and 186.1579, corresponding to $C_{11}H_{19}O_2^-$ and D_3-$C_{11}H_{16}O_2^-$, from *endo*-metabolome extracts of *hacl-1(tm6725)* larvae supplemented with Met or D_3-Met. Under these chromatographic conditions, bemeth#1 elutes as a shoulder of an unidentified isobaric compound (undecenoic acid). Blue box highlights D_3-enrichment in later-eluting bemeth#1, which was resolved from the earlier metabolite via method optimization (see **Fig. 3B**).

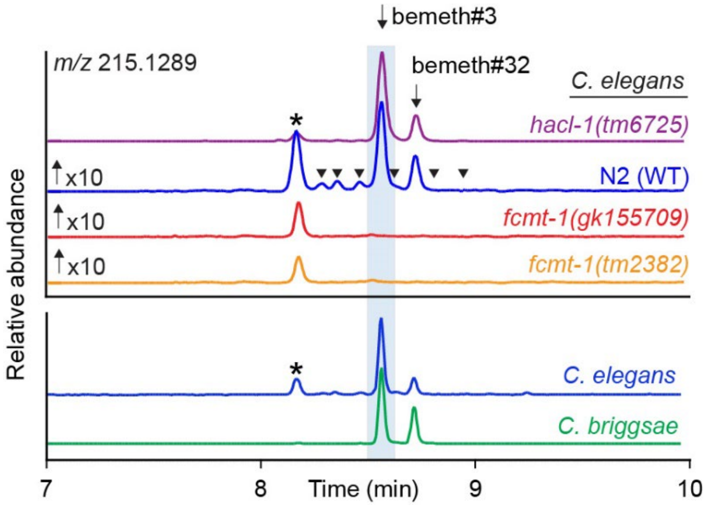

Supplementary Fig. 8. βMFAs are detected in *C. briggsae*.

EICs for *m/z* 215.1289, corresponding to bemeth#3 and isomers of $C_{11}H_{19}O_4^-$, in extracts of *hacl-1(tm6725),* N2 (WT), *fcmt-1(gk155709)*, and *fcmt-1(tm2382)*, as well as in independently grown *C. elegans* and *C. briggsae* cultures, as indicated. bemeth#3 and its stereoisomer (bemeth#32) are marked with arrows and detected in *C. elegans* and *C. briggsae*, enriched in *hacl-1* but not detected in *fcmt-1* mutants. An unrelated FCMT-1-independent isomer is marked with an asterisk (*). Additional minor isomers in *C. elegans* are marked with arrowheads. Y-axes are scaled as indicated to clearly show traces.

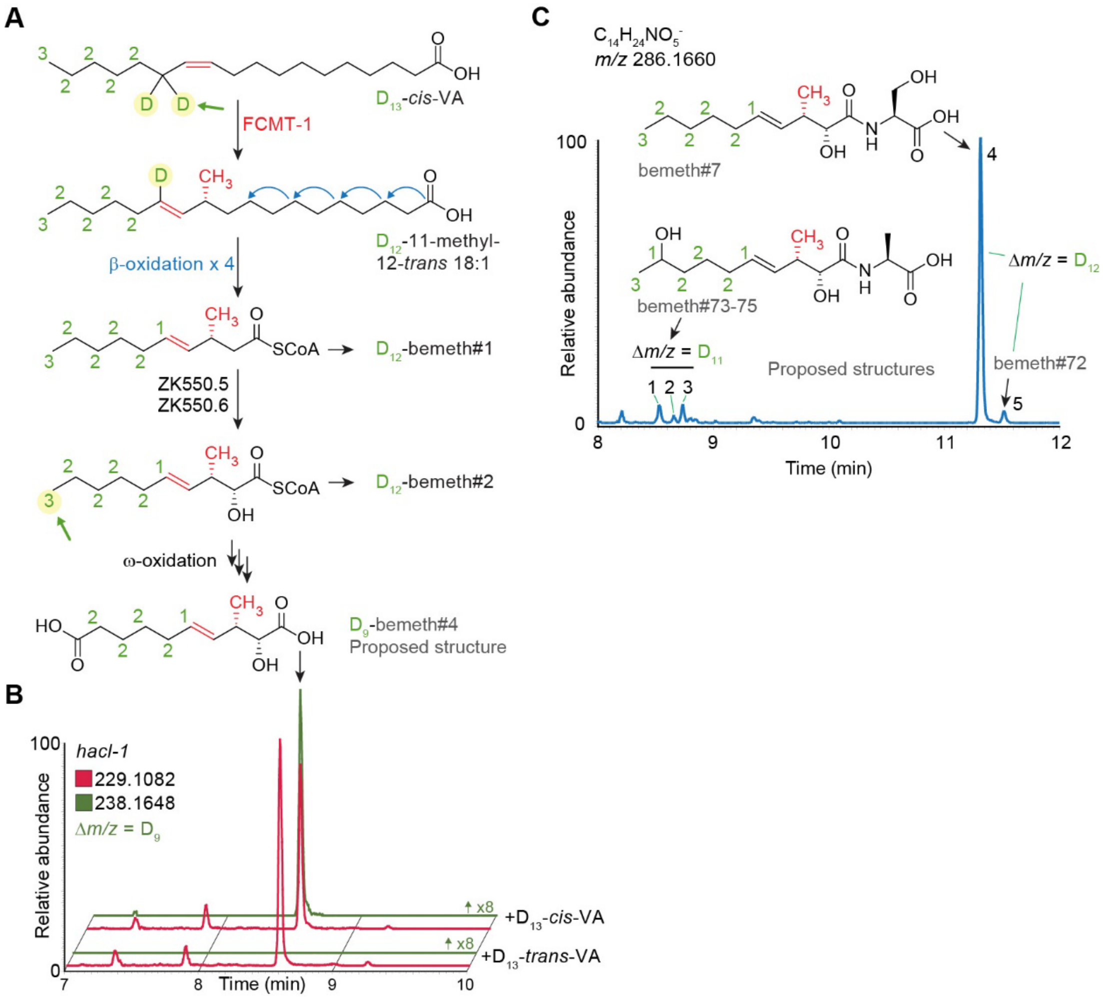

Supplementary Fig. 9. Isotopic enrichment from *cis*-D_{13}-vaccenic acid supplement

A) Proposed biosynthesis of bemeth#1 based on isotopically labeled D_{13}-VA feeding experiment (see **Fig. 5B**). The number of deuterium atoms on each carbon is labeled green. Methyl transfer results in abstraction of one deuterium atom (highlighted with green arrow) and distal oxidation (highlighted with green arrow) results in metabolites with a diagnostic number of deuterium atoms remaining.

B) EICs for *m/z* 229.1082 and 238.1648, corresponding to bemeth#4 and D_9-bemeth#4, respectively, in extracts of *hacl-1* mixed-stage cultures supplemented with D_{13}-*cis*- or D_{13}-*trans*-VA. Oxidation of the ω-carbon results in the loss of three additional deuterium atoms, for a total loss of four deuterium atoms and a diagnostic isotope label. Y-axis for *m/z* 238.1648 is scaled 8-fold to clearly show traces for labelled features.

C) EIC for *m/z* 286.1660, corresponding to $C_{14}H_{25}NO_5^-$, in extract of N2 (WT) mixed-stage culture supplemented with D_{13}-*cis*-VA. Earlier eluting isomers are D_{11}-enriched (bemeth#73-75), whereas later eluting isomers are D_{12}-enriched (bemeth#7, bemeth#72). Representative structures proposed based on isotope labeling and MS/MS fragmentation.

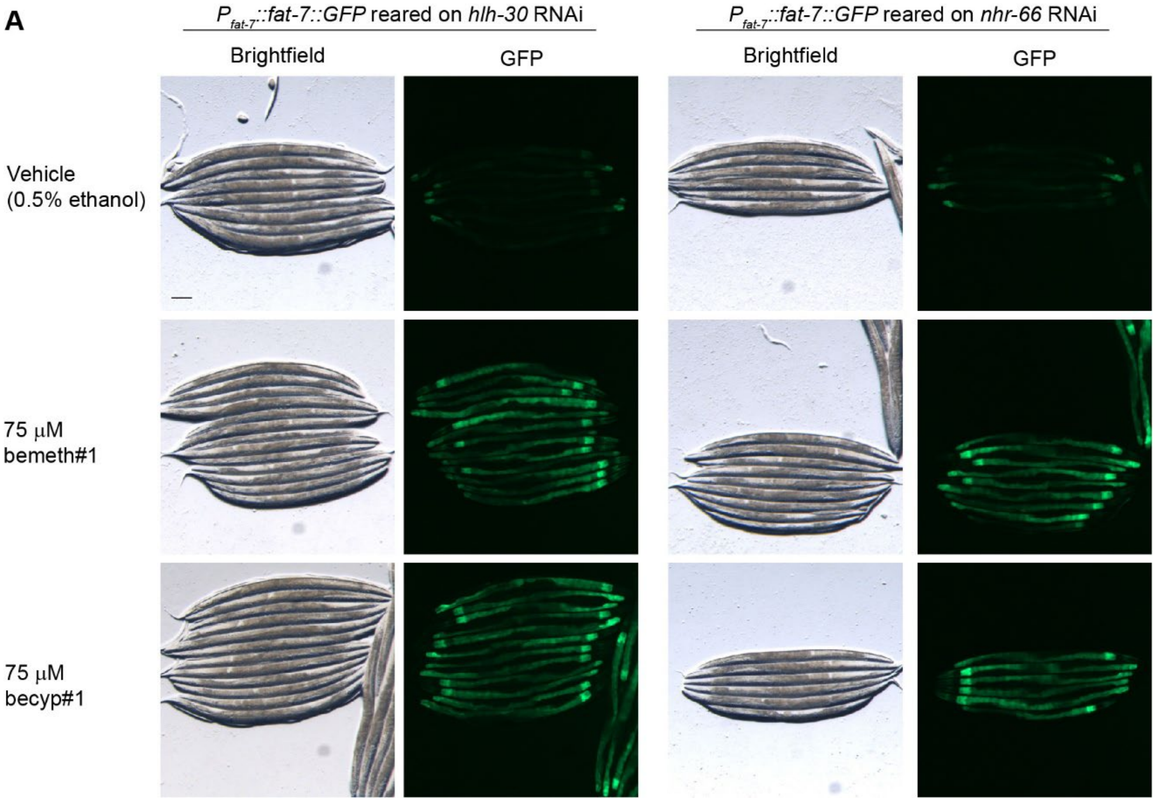

Supplementary Fig. 10. FAT-7::GFP induction is independent of *hlh-30* and *nhr-66*.

A) Representative brightfield and fluorescence micrographs of $P_{fat\text{-}7}$::*fat-7::GFP* animals reared on HT115 expressing *hlh-30* or *nhr-66* RNAi and supplemented with vehicle only (0.5% ethanol), 75 μM bemeth#1, or 75 μM becyp#1. Animals were supplemented in parallel with animals reared on HT115 expressing L4440 (vector control), see **Fig. 5**. Scale bar represents 0.1 mm. Five independent experiments were performed.

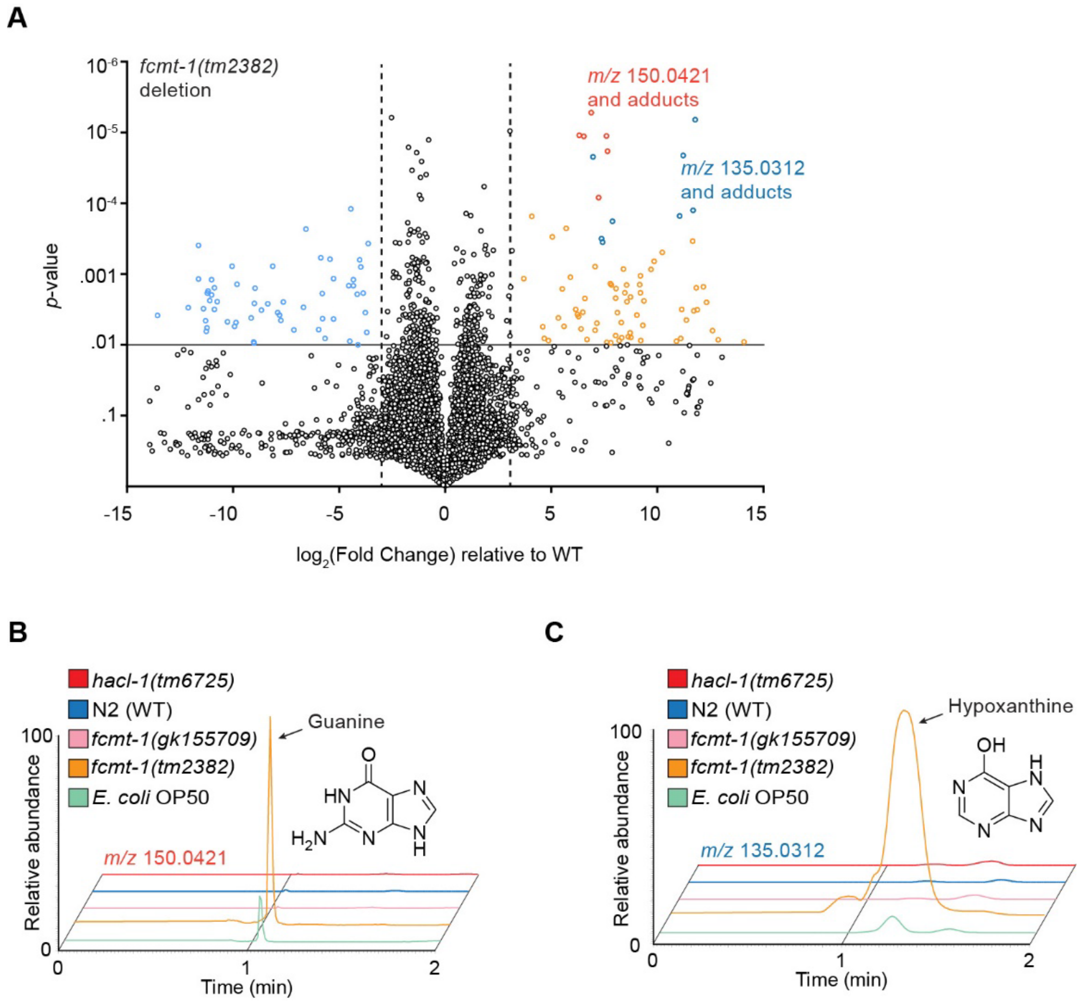

Supplementary Fig. 11. Unique metabolites enriched in *fcmt-1(tm2382)*

A) Volcano plot for subset of features detected by HPLC-MS (negative ion) in the *exo*-metabolome of *fcmt-1(tm2382)* relative to wildtype (N2) control. *P*-values were calculated by unpaired, two-sided Welch's t-test; no adjustments were made for multiple comparisons. Red points represent *m/z* 150.0421, including isotopes and adducts, corresponding to guanine; blue points represent *m/z* 135.0312, corresponding to hypoxanthine. Both metabolites were confirmed by commercial standards.

B) Representative EICs for *m/z* 150.0421, corresponding to guanine, in *exo*-metabolome extracts of synchronized adult N2 (WT), *hacl-1(tm6725), fcmt-1(gk155709),* and *fcmt-1(tm2382)* animals, or from extract of *E. coli* OP50 only (bacterial diet).

C) Representative EICs for *m/z* 135.0312, corresponding to hypoxanthine, in *exo*-metabolome extracts of synchronized adult N2 (WT), *hacl-1(tm6725), fcmt-1(gk155709),* and *fcmt-1(tm2382)* animals, or from extract of *E. coli* OP50 only (bacterial diet).

Supplementary Methods

General synthetic procedures.

Unless stated otherwise, all reactions were carried out under argon (Ar) atmosphere in flame-dried glassware. All commercially available reagents were used as purchased unless otherwise stated. All solvents were dried over activated 3Å molecular sieves for a minimum of 24 hours unless used in reactions containing aqueous reagents. Solutions and solvents sensitive to moisture and oxygen were transferred via standard syringe and cannula techniques. Reactions were cooled with ice-water or dry ice-acetone baths or heated with mineral oil baths depending on reaction temperature. Titanium (IV) isopropoxide was distilled under vacuum and stored under argon. Thin-layer chromatography (TLC) was performed with J.T. Baker Silica Gell IB2-F plastic-backed plates with analysis via UV and p-anisaldehyde stain. Flash chromatography was performed using Teledyne ISCO CombiFlash Rf and Rf+ systems with Teledyne ISCO RediSep Rf and Rf Gold silica columns. All deuterated solvents were purchased from Cambridge Isotopes. Nuclear Magnetic Resonance (NMR) spectra were recorded on a Varian INOVA 600 (600 MHz) or Bruker AV 500 (500 MHz) in the Cornell University NMR Facility. ^{1}H NMR chemical shifts are reported in ppm (δ) relative to the residual solvent peaks (7.26 ppm for $CDCl_3$ and 3.31 ppm for CD_3OD) and ^{13}C NMR shifts relative to their respective residual solvent peaks (77.16 for $CDCl_3$ and 49.00 for CD_3OD). NMR-spectroscopic data are reported as follows: chemical shift, multiplicity (s = singlet, d = doublet, t = triplet, q = quartet, m = multiplet, br = broad), coupling constants (Hz), and integration and often tabulated including 2D NMR data. All NMR data processing was done using MNOVA 12.0.1 (https://mestrelab.com/).

Chemical syntheses

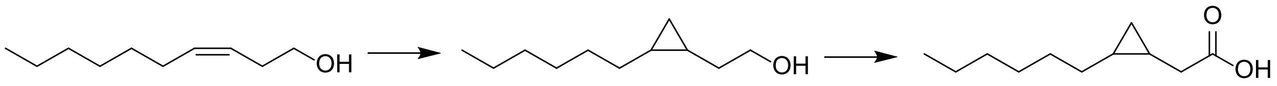

(**1**) (**2**) becyp#1

***cis*-3,4-methylenedecanoic acid, becyp#1.** From an 8:1 cis:trans solution of **1** (40 mg, 0.256 mmol, 1.00 equiv.) in 1.5 mL DCM was added $Zn(Et)_2$ (1M in hexanes, 1.28 mL, 1.28 mmol, 5.00 equiv.). The solution was cooled to 0 ˚C followed by the addition of CH_2I_2 (102 µL, 1.28 mmol, 5.00 equiv.). The resulting suspension was allowed to reach room temp with stirring and remained at room temp. overnight. The yellow mixture was directly purified via flash column chromatography on silica gel using a gradient of 5-100% EtOAc in hexanes, affording cyclopropyl alcohol intermediate (**2**, 40 mg) with some uncharacterized impurities. To a solution of the cyclopropyl alcohol intermediate (**2**, 10 mg) in 1 mL of acetone at 0 ˚C was added 6 drops of freshly prepared Jones reagent (enough to maintain an orange color). The solution was stirred at 0 ˚C for 1 hr and then directly purified via flash column chromatography on silica gel using a gradient of 5-100% EtOAc in hexanes, affording becyp#1 (8 mg, 69% over two steps) as a colorless oil, determined as a mixture of 8:1 *cis : trans* isomers.

^{1}H NMR (600 MHz, chloroform-*d*): δ 2.42 (dd, *J* = 16.6, 7.0 Hz, 1H), 2.30 (dd, *J* = 16.6, 7.6 Hz, 1H), 1.44 – 1.20 (m, 10H), 1.15 (m, 1H), 1.09 (m, 1H), 0.88 (t, *J* = 6.8 Hz, 3H), 0.74 (td, *J* = 8.4, 4.6 Hz, 1H), -0.11 (q, *J* = 5.2 Hz, 1H). These chemical shifts were nearly identical to those previously reported[1].

^{13}C NMR (126 MHz, chloroform-*d*): δ 180.1, 33.8, 32.2, 30.0, 29.4, 28.9, 22.8, 15.6, 14.2, 11.3, 11.

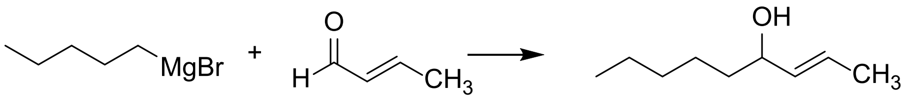

(**3**) (**4**) (**5**)

***(E)*-4-Hydroxy-non-2-ene (5).** *n*-Pentylmagnesium bromide (**3**, 10 mL, 20 mmol in THF) was added to a diethyl ether (20mL) at 0 °C under an argon atmosphere. Crotonaldehyde (**4**, 1.66 mL, 20 mmol) was added to the stirring solution over 10 minutes via an addition funnel and stirred for one hour. The reaction was quenched with saturated aqueous NH4Cl (20 mL) and extracted with EtOAc (3×20 mL). The combined organics were dried over $MgSO_4$, filtered, and concentrated under reduced pressure. The resulting liquid was purified by flash chromatography on silica gel. Elution with a gradient of 0-20% EtOAc/Hexanes gave the resulting alcohol (**5**) (2.61 g, 92%) as a colorless liquid[2].

^{1}H NMR (CDCl3, 500 MHz): δ (ppm) 5.65 (dqd 15.3, 6.5, 0.8 Hz, 1H), 5.48 (ddq 15.3, 7.7, 1.5 Hz, 1H), 4.03 (q, 6.6 Hz, 1H), 1.70 (dd 6.5, 1.3 Hz, 3H), 1.25-1.58 (m, 9H), 0.89 (t, 6.8 Hz, 3H).
^{13}C NMR (CDCl3, 125 MHz): δ (ppm) 134.5, 126.9, 73.4, 37.4, 31.9, 25.3, 22.8, 17.8, 14.2.
(**2**) **(ESI)** ***m/z*****:** Calculated: $(M+H)^+$ 143.1430. Actual: 143.1428 Δ ppm: -1.64

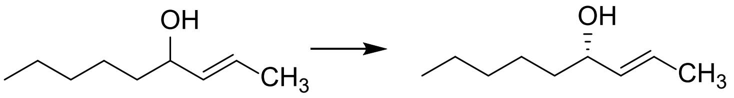

(**5**) (*S*)-(**5**)

(S,E)**-4-Hydroxy-non-2-ene (*****(S)*****-5).** *(-)*-Diisopropyl D-tartrate (183 µL, 0.88 mmol) was added to a suspension of powdered 4 Å molecular sieves (0.4 g) in DCM (21 mL) at ambient temperature under an argon atmosphere. The solution was cooled to -20 °C and titanium (IV) isopropoxide (212 µL, 0.7 mmol) was added. The solution was stirred for one hour and *tert*-butylhydroperoxide (382 µL, 2.1 mmol in decane) added. The solution was further stirred for 30 minutes and cooled to -40 °C, upon which racemic *(E)*-4-hydroxy-non-2-ene (**5**) (3.5 mL, 3.5 mmol in DCM) was added dropwise. After 20 hours the reaction was quenched with *(-)*-diisopropyl D-tartrate (366 µL, 1.75 mmol) in water (7 mL). The layers were separated, and the aqueous solution extracted with Et_2O (3×20 mL). The combined organics were washed with saturated aqueous $NaHCO_3$, dried over $MgSO_4$, filtered, and concentrated under reduced pressure. The resulting liquid was purified by flash chromatography on silica gel as above, yielding chiral alcohol *(S)*-**5** (236 mg, 94%) as a colorless liquid[3]. Enantiomeric excess was determined to be up to 88% by Mosher derivatization[4]. All NMR spectra and mass spectrometric data are identical to racemic alcohol (**5**).

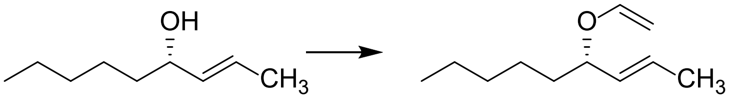

(*S*)-(**5**) (*S*)-(**6**)

(S,E)**-4-(Vinyloxy)-non-2-ene (*****(S)*****-6).** *(S,E)*-4-Hydroxy-non-2-ene (*(S)*-**5**, 307 mg, 2.16 mmol) was added to ethyl vinyl ether (5.75 mL, 60.5 mmol) at ambient temperature under an argon atmosphere. Mercury (II) acetate (688 mg, 2.16 mmol) was added to the solution[5]. After two hours AcOH (302 µL) was added with stirring. After 30 minutes the reaction was diluted with hexanes (45 mL) and washed with 5% aqueous KOH (4.5 mL). The organics were dried over $MgSO_4$, filtered, and concentrated under reduced pressure. The resulting liquid was purified by flash chromatography on alumina. Elution with a gradient of 0-10% EtOAc/Hexanes yielded vinyl ether (*(S)*-**6**, 178 mg, 76% BRSM) as a colorless liquid.

^{1}H NMR ($CDCl_3$, 500 MHz) δ (ppm) 6.31 (dd, 14.1, 6.6 Hz, 1H), 5.65 (dqd 15.3, 6.6, 0.8 Hz, 1H), 5.38 (ddq, 15.3, 7.6, 1.6 Hz, 1H), 4.28 (dd, 14.1, 1.4 Hz, 1H), 4.05 (q, 7 Hz, 1H), 3.96 (dd, 6.6, 1.4 Hz, 1H), 1.71 (dd, 6.5, 1.4 Hz, 3H), 1.61-1.70 (m, 1H), 1.46-1.54 (m, 1H), 1.23-1.40 (m, 6H), 0.88 (t, 6.8 Hz, 3H).

^{13}C NMR ($CDCl_3$, 125 MHz) δ (ppm) 151.0, 131.4, 128.7, 88.4, 81.2, 35.3, 31.8, 25.0, 22.7, 17.9, 14.2.

HRMS (ESI) *m/z*: Calculated: $(M+H)^+$ 169.1587 $(M+Na)^+$ 191.1406. Actual: 169.1584 Δ ppm: -1.99

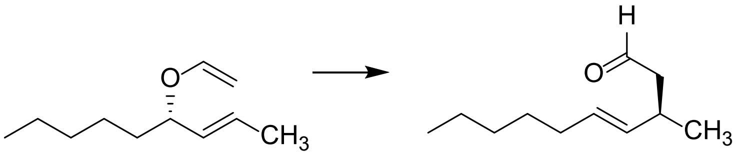

(*S*)-(**6**) (*R*)-(**7**)

***(R,E)*-3-Methyl-dec-4-enal (*(R)*-7).** Vinyl ether (*(S)*-**6**, 107 mg, 0.64 mmol) was added to toluene (5 mL) and stirred with condenser at reflux under an argon atmosphere. After 23 hours the reaction was concentrated to yield aldehyde (*(R)*-**7**, 100 mg, 93%) as a colorless liquid which was used without purification[5–7].

^{1}H NMR (CDCl3, 500 MHz) δ (ppm) 9.72 (t, 2.4 Hz, 1H), 5.44 (dtd, 15.4, 6.6, 1.0 Hz, 1H), 5.34 (ddt, 15.4, 7.0, 1.3 Hz, 1H), 2.72 (m, 6.9 Hz, 1H), 2.40 (ddd, 16.0, 7.3, 2.4 Hz, 1H), 2.33 (ddd, 16.0, 6.7, 2.4 Hz, 1H), 1.97 (q, 7.0 Hz, 2H), 1.21-1.37 (m, 6H), 1.06 (d, 6.8 Hz, 3H), 0.88 (t, 7.0 Hz, 3H).

^{13}C NMR (CDCl3, 125 MHz) δ (ppm) 203.0, 133.9, 130.2, 50.7, 32.6, 31.8, 31.5, 29.3, 22.7, 20.9, 14.2.

HRMS (ESI) *m/z*: Calculated: $(M+Na)^+$ 191.1406. Actual: 191.1411 Δ ppm: 2.49

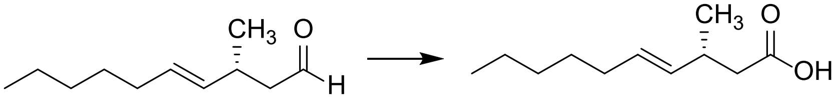

(*R*)-(**7**) (*R*)-bemeth#1

***(R,E)*-3-Methyl-dec-4-enoic acid, *(R)*-bemeth#1.** Aldehyde (*(R)*-**13**, 53 mg, 0.32 mmol) was dissolved in DMSO (1.1 mL) and stirred under ambient atmosphere. Sodium chlorite (40 mg, 0.35 mmol) was dissolved in minimal water and the pH adjusted to ~4.5 with NaH_2PO_4. The aqueous solution was added to the aldehyde and the reaction stirred in an open atmosphere. After 30 minutes, more sodium chlorite was added to the reaction (20 mg, 0.18 mmol) dissolved in water and buffered as above. After 45 minutes the reaction was diluted with water (2 mL) and extracted

with EtOAc (3×2 mL). The combined organics were dried over $MgSO_4$, filtered, and concentrated under reduced pressure. The resulting oil was purified by flash chromatography on silica gel. Elution with a gradient of 0-20% EtOAc(0.1% AcOH)/hexanes yielded *(R)*-bemeth#1 (**4**, 44 mg, 76%) as a colorless oil. Enantiomeric excess was determined to be 65% by 2,2,2- trifluoro-1-phenethylamine derivatization and subsequent analysis by UHPLC-MS.

^{1}H NMR (CDCl3, 500 MHz) δ (ppm) 5.45 (dtd, 15.4, 6.7, 0.8 Hz, 1H), 5.33, ddt, 15.3, 7.2, 1.3, 1H), 2.63 (m, 7.0 Hz, 1H), 2.35 (dd, 14.9, 7.3 Hz, 1H), 2.28 (dd, 14.9, 7.3 Hz, 1H), 1.96 (q, 7.0, 2H), 1.21-1.36 (m, 6H), 1.05 (d, 6.7 Hz, 3H), 0.88 (t, 6.9 Hz, 3H).

^{13}C NMR (CDCl3, 125 MHz) δ (ppm) 178.8, 133.7, 1.0.1, 41.8, 33.5, 32.6, 34.4, 29.3, 22.7, 20.5, 14.2.

HRMS (ESI) *m/z*: Calculated: $(M-H)^-$ 183.1391. Actual: 183.1383. Δ ppm: -4.16.

***(S,E)*-3-Methyl-dec-4-enoic acid, *(S)*-bemeth#1.** Following Sharpless resolution using *(+)*-Diisopropyl L-tartrate, parallel reactions yielded *(S)*-bemeth#1 with identical physical and spectroscopic properties.

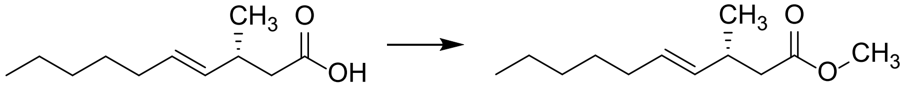

(*R*)-bemeth#1 (*R*)-(**8**)

Methyl-*(R, E)*-3-methyl-dec-4-enoate (*(R)*-8). Trimethylsilyldiazomethane (1.2 mL, 0.72 mmol) was added dropwise to a solution of *(R)*-bemeth#1 in DCM (3 mL) and MeOH (3 mL). After stirring for 15 minutes, the reaction was concentrated under reduced pressure. The resulting oil was purified by flash chromatography on silica gel. Elution with a gradient of 0- 10% EtOAc/hexanes yielded methyl ester (*(R)*-**8**, 42 mg, 89%) as a colorless oil.

^{1}H NMR (CDCl3, 500 MHz) δ (ppm) 5.42 (dtd, 15.3, 6.7, 0.9 Hz, 1H), 5.31 (ddt, 15.3, 7.3, 1.2 Hz, 1H), 3.63 (s, 3H), 2.61 (m, 7.0 Hz, 1H), 2.30 (dd, 14.7, 7.3 Hz, 1H), 2.24 (dd, 14.7, 7.3 Hz, 1H), 1.95 (q, 6.9 Hz, 2H), 1.21-1.35 (m, 6H), 1.02 (d, 6.9 Hz, 3H), 0.87 (t, 6.9 Hz, 3H).

^{13}C NMR (CDCl3, 125 MHz) δ (ppm) 173.3, 134.0, 129.8, 51.5, 42.0, 33.8, 32.6, 31.4, 29.3, 22.7, 20.6, 14.

HRMS (ESI) *m/z*: Calculated: $(M+H)^+$ 199.1693. Actual: 199.1690. Δ ppm: -1.49.

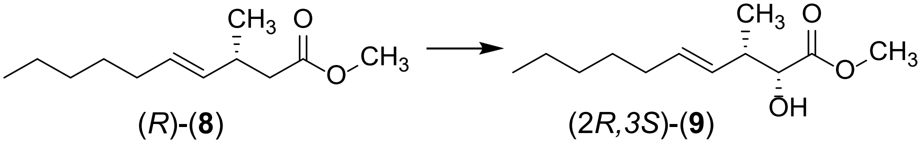

(*R*)-(**8**) → (2*R*,3*S*)-(**9**)

Methyl-*(2R,3S,E)*-2-hydroxy-3-methyl-dec-4-enoate (*(2R,3S)*-9). *n*-Butyl lithium (72 µL, 0.18 mmol) was added dropwise to a stirring solution of *N,N*-diisopropylamine (21 µL, 0.15 mmol) in THF (4 mL) at -15°C and stirred 10 minutes under an argon atmosphere. The solution was cooled to -78°C and methyl ester (*(R)*-**8**) was added and the reaction stirred at -15°C for 15 minutes. The reaction was cooled to -78°C and *(+)*-(8, 8-dichlorocamphorylsulfonyl) oxaziridine (90 mg, 0.3 mmol) was added in THF (2 mL) and stirred to -15°C[8]. After stirring for one hour, the reaction was quenched with aqueous saturated $NaHCO_3$ (3 mL) and the layers separated. The aqueous layer was extracted with DCM (3×5mL) and the combined organics dried over $MgSO_4$, filtered, and concentrated under reduced pressure. The resulting oil was purified by flash chromatography on silica gel. Elution with a gradient of 0-20% EtOAc/hexanes yielded alpha-hydroxy ester (*(2R,3S)*-**9**, 9 mg, 69% BRSM).

^{1}H NMR (CDCl3, 500 MHz) δ (ppm) 5.53 (dtd, 15.3, 6.7, 0.9 Hz, 1H), 5.39 (ddt, 15.3, 7.9, 1.4 Hz, 1H), 4.12 (d, 4.2, 1H), 3.78 (s, 3H), 3.76 (q, 4.3 Hz, 1H), 2.52-2.67 (m, 1H), 2.00 (qd, 7.1, 1.2 Hz, 2H), 1.21-1.38 (m, 8H), 0.99 (d, 7.0, 3H), 0.88 (t, 6.9, 3H).

^{13}C NMR (CDCl3, 125 MHz) δ (ppm) 174.8, 132.4, 130.6, 74.6, 52.4, 41.3, 32.7, 31.5, 29.2, 22.7, 14.8, 14.2.

HRMS (ESI) *m/z*: Calculated: $(M+Na)^+$ 237.1461. Actual: 237.1475. Δ ppm: 5.87.

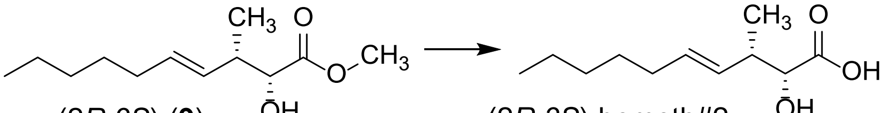

(2*R*,3*S*)-(**9**) → (2*R*,3*S*)-bemeth#2

***(2R,3S,E)*-2-hydroxy-3-methyl-dec-4-enoic acid, *(2R,3S)*-bemeth#2.** Lithium hydroxide (40 mg, 2 mmol) was added to a stirring solution of ester (*(2R,3S)*-**9**, 11 mg, 0.05 mmol) in MeOH (0.4 mL), THF (0.4 mL), and water (0.2 mL). After one hour, the reaction was acidified with 1 M HCl and extracted with DCM (3×5 mL). The combined organics were dried over MgSO4, filtered, and concentrated under reduced pressure. The resulting oil was purified by flash chromatography on silica gel. Elution with a gradient of 0-100% DCM/MeOH(0.1% AcOH) yielded alpha-hydroxy acid *(2R,3S)*-bemeth#2 (8.6 mg, 86% BRSM, d.r. 67.8% as determined by Mosher analysis[9]).

^{1}H NMR (CDCl3, 500 MHz) δ (ppm) 5.58 (dt, 15.4, 6.8 Hz, 1H), 5.42 (dd, 15.4, 7.6 Hz, 1H), 4.22 (d, 3.6, 1H), 2.68 (m, 1H), 2.02 (q, 6.9 Hz, 2H), 1.22-1.40 (m, 6H), 1.05 (d, 7.0, 3H), 0.89 (t, 6.7, 3H).

^{13}C NMR (CDCl3, 125 MHz) δ (ppm) 177.4, 133.1, 130.1, 74.2, 40.7, 32.7, 31.5, 29.2, 22.7, 14.3, 14.2

HRMS (ESI) *m/z*: Calculated: $(M\text{-}H)^-$ 199.1340. Actual: 199.1339. Δ ppm: -0.28.

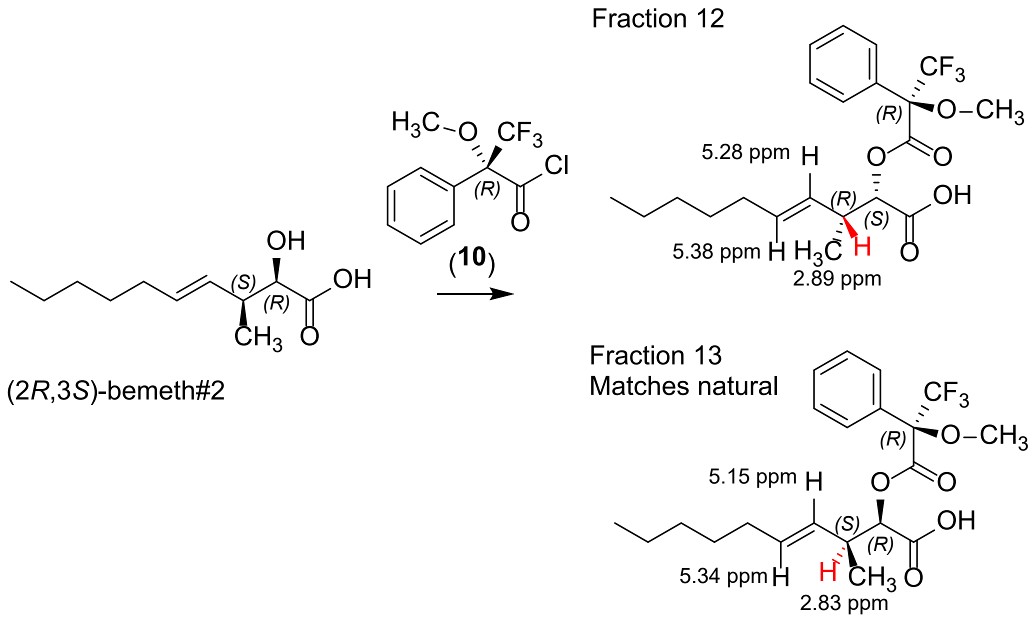

Determination of bemeth#2 stereochemistry. bemeth#2 was dissolved in DCM with DMAP and stirred under argon at ambient temperature. *(R)*-(+)-α-Methoxy-α-(trifluoromethyl)phenylacetyl chloride (**10**, 1.2 equivalents) was added and the reaction stirred for 30 minutes and quenched with MeOH. The reaction was concentrated under reduced pressure, taken up in MeOH, and analyzed by HPLC-HRMS. Integration of the EIC after Mosher derivatization of synthetic bemeth#2 yielded a diastereomeric enrichment of 68%.

Supplementary Tables

Supplementary Table 1. Metabolites enriched in *acdh-11(n5878)* mutants. Subset of dereplicated metabolites that are i) at least 8-fold enriched in *acdh-11(n5878);P*$_{fat\text{-}7}$*::fat-7::GFP* relative to WT *P*$_{fat\text{-}7}$*::fat-7::GFP,* ii) mean intensity > 500,000 AU for *acdh-11*, iii) not detected in bacteria only, and iv) dependent on cyclopropane lipid biosynthesis in *E. coli*. These data were filtered using stringent criteria and hundreds of additional differential features were detected (not dereplicated) using mean intensity cutoff at 100,000 AU. Note: these metabolites were detected in *acdh-11(n5878)* animals reared on OP50, HB101, and BW25113.

ES(-) Obs. *m/z*	RT (min)	Molecular Formula	ES(-) Theor. *m/z*	*m/z* error (ppm)	SMID-DB #	Comments
242.10394	6.90	$C_{11}H_{17}NO_5$	242.10340	2.226		
171.06656	6.96	$C_8H_{12}O_4$	171.06628	1.592		likely dicarboxylate, Na adduct in ES(-)
457.14896	7.14	$C_{17}H_{31}O_{12}P$	457.14804	2.013		endo
574.20697	7.19	$C_{25}H_{38}NO_{12}P$	574.20589	1.881		endo
576.22237	7.32	$C_{25}H_{40}NO_{12}P$	576.22154	1.449		endo
396.17978	7.47	$C_{16}H_{32}NO_8P$	396.17928	1.261		endo
427.13823	7.62	$C_{16}H_{28}O_{11}P$	427.13747	1.769		
215.12915	7.75	$C_{11}H_{20}O_4$	215.12888	1.225	becyp#32	
229.10845	7.85	$C_{11}H_{18}O_5$	229.10815	1.331		
215.12920	7.94	$C_{11}H_{20}O_4$	215.12888	1.483	becyp#33	
185.08227	7.95	$C_9H_{14}O_4$	185.08193	1.797		likely dicarboxylate, Na adduct in ES(-)
227.09299	8.02	$C_{11}H_{16}O_5$	227.09250	2.131		
213.11365	8.12	$C_{11}H_{18}O_4$	213.11323	1.979		*endo*
576.22271	8.15	$C_{25}H_{40}NO_{12}P$	576.22154	2.029		*endo*
213.11378	8.16	$C_{11}H_{18}O_4$	213.11323	2.591		
215.12918	8.18	$C_{11}H_{20}O_4$	215.12888	1.360	becyp#3	
213.11380	8.18	$C_{11}H_{18}O_4$	213.11323	2.662		
439.13818	8.27	$C_{17}H_{28}O_{11}P$	439.13747	1.600		*endo*, MOGL
229.10852	8.30	$C_{11}H_{18}O_5$	229.10815	1.630		
286.16662	8.31	$C_{14}H_{25}NO_5$	286.16600	2.179		
441.15367	8.35	$C_{17}H_{30}O_{11}P$	441.15312	1.239		*endo, MOGL*
286.16672	8.38	$C_{14}H_{25}NO_5$	286.16600	2.528		formate adduct
276.13761	8.38	$C_{13}H_{24}NO_3Cl$	276.13720	1.509		
288.18226	8.41	$C_{14}H_{27}NO_5$	288.18165	2.143		formate

						adduct
213.11378	8.47	$C_{11}H_{18}O_4$	213.11323	2.591		*endo*
591.17201	8.49	$C_{22}H_{32}N_4O_{13}P$	591.1708979	1.878		*endo*
441.15376	8.54	$C_{17}H_{30}O_{11}P$	441.15312	1.451		*endo*
254.14035	8.61	$C_{13}H_{21}NO_4$	254.13978	2.243		
405.17729	8.62	$C_{18}H_{29}O_{10}$	405.17662	1.652		formate adduct
256.15597	8.63	$C_{13}H_{23}NO_4$	256.15543	2.092		
361.18767	8.70	$C_{17}H_{30}O_8$	361.18680	2.444		
199.09793	8.88	$C_{10}H_{16}O_4$	199.09758	1.747		likely dicarboxylate, Na adduct in ES(-)
716.30686	9.12	$C_{33}H_{52}NO_{14}P$ (?)	716.30527	2.226		endo
270.17162	9.13	$C_{14}H_{25}NO_5$	270.17108	1.999		
758.35478	9.42	$C_{36}H_{57}NO_{14}P$ (?)	758.35222	3.383		endo
756.33820	9.44	$C_{36}H_{55}NO_{14}P$	756.33657	2.165		endo
560.22734	9.58	$C_{25}H_{40}NO_{11}P$	560.22662	1.285		endo
199.13434	9.77	$C_{11}H_{20}O_3$	199.13397	1.850	becyp#2	characterized by 2D-NMR
213.11365	9.79	$C_{11}H_{18}O_4$	213.11323	1.970	becyp#4	likely dicarboxylate, Na adduct in ES(-)
560.19103	9.89	$C_{24}H_{36}NO_{12}P$	560.19024	1.420		MOGL
197.11851	9.90	$C_{11}H_{18}O_3$	197.11832	0.988		endo
558.17604	9.93	$C_{24}H_{34}NO_{12}P$	558.17459	2.603		
542.23839	9.96	$C_{22}H_{42}NO_{12}P$	542.23719	1.864		GLEA
199.13420	10.02	$C_{11}H_{20}O_3$	199.13397	1.183	becyp#22	endo, minor
639.27995	10.36	$C_{28}H_{48}O_{14}P$	639.27871	1.928		endo
411.14319	10.47	$C_{16}H_{28}O_{10}P$	411.14255786	1.533		endo
560.19090	10.57	$C_{24}H_{36}NO_{12}P$	560.19024	1.179		endo, MOGL, anthranilate
558.17534	10.66	$C_{24}H_{34}NO_{12}P$	558.17459	1.345		endo
584.19113	10.68	$C_{26}H_{35}NO_{12}P$	584.19023	1.537		endo
540.20149	10.74	$C_{25}H_{36}NO_{10}P$	540.20041	2.009	iglu#202	MOGL
411.14318	10.80	$C_{16}H_{28}O_{10}P$	411.14256	1.512		endo
377.18250	11.16	$C_{17}H_{30}O_9$	377.18171	2.100		formate adduct
544.19667	12.58	$C_{24}H_{36}NO_{11}P$ (?)	544.19532	2.468		
335.17716	17.31	$C_{21}H_{24}N_2O_2$	335.17650	1.950		
183.13906	9.95	$C_{11}H_{20}O_2$	183.13905	0.037	becyp#1	post-column ion pairing

Supplementary Table 2. FCMT-1-derived metabolites. A comprehensive list of FCMT-1-derived metabolites that were detected in the conditioned media (*exo*-) or worm body *(endo-)* metabolome of N2 (WT) synchronized gravid adults in liquid culture, unless otherwise indicated.

ES(-) Obs. *m/z*	RT (min)	Molecular Formula	ES(-) Theor. *m/z*	*m/z* error (ppm)	SMID-DB #	Comments
231.12393	7.18	$C_{11}H_{20}O_5$	213.12380	0.584	bemeth#401	enriched *endo*, H/D exchange $D_8/D_9/D_{10}$
215.12902	8.33	$C_{11}H_{20}O_4$	215.12888	0.725	bemeth#33	minor, D_{11}
215.12902	8.40	$C_{11}H_{20}O_4$	215.12888	0.725	bemeth#34	minor, D_{11}
215.12900	8.51	$C_{11}H_{20}O_4$	215.12888	0.545	bemeth#35	minor, D_{11}
286.16622	8.57	$C_{14}H_{25}NO_5$	286.16600	0.785	bemeth#73	D_{11}
213.11345	8.58	$C_{11}H_{18}O_4$	213.11323	1.014	bemeth#322	enriched *endo*, H/D exchange $D_5/D_6/D_7$
215.12904	8.61	$C_{11}H_{20}O_4$	215.12888	0.731	bemeth#3	major, D_{11} presumed 2*R*,3*S*
229.10833	8.65	$C_{11}H_{18}O_5$	229.10815	0.778	bemeth#4	enriched *hacl*-1, D_9
286.16620	8.69	$C_{14}H_{25}NO_5$	286.16600	0.711	bemeth#74	D_{11}
286.16618	8.76	$C_{14}H_{25}NO_5$	286.16600	0.641	bemeth#75	D_{11}
215.12901	8.78	$C_{11}H_{20}O_4$	215.12888	0.616	bemeth#32	secondary, D_{11}
546.21123	8.80	$C_{24}H_{38}NO_{11}P$	546.21097	0.478	TBD	detected only in N2, D_{12}
231.12395	8.93	$C_{11}H_{20}O_5$	213.12380	0.684	bemeth#402	detected only in *hacl-1(tm6725)*
215.12894	9.01	$C_{11}H_{20}O_4$	215.12888	0.266	bemeth#37	minor, D_{11}
361.18722	9.08	$C_{17}H_{30}O_8$	361.18679	1.194	bemeth#8	putative glucoside
591.17185	9.13	$C_{22}H_{33}N_4O_{13}P$	591.17090	1.615	gluric#421	MOGL, enriched *endo*, co-eluting cyclopropyl isomer, D_{11}
215.12885	9.14	$C_{11}H_{20}O_4$	215.12888	0.152	bemeth#38	minor, D_{11}
576.22211	9.21	$C_{25}H_{39}NO_{12}P$	576.22154	1.003	oglu#421	MOGL, *endo*
591.17178	9.26	$C_{22}H_{33}N_4O_{13}P$	591.17090	1.506	gluric#422	MOGL, enriched *endo*, D_{11}
607.16641	9.28	$C_{22}H_{33}N_4O_{14}P$	607.16581	0.984	gluric#431	MOGL, enriched *endo*
268.15553	9.28	$C_{14}H_{23}NO_4$	268.15543	0.369	bemeth#622	
441.15345	9.29	$C_{17}H_{31}O_{11}P$	441.15312	0.747	bemeth#82	putative phosphorylated glulcoside, *endo*
558.21201	9.64	$C_{25}H_{38}NO_{11}P$	558.21097	1.854	TBD	D_{12}
213.11343	10.06	$C_{11}H_{18}O_4$	213.11323	0.529	bemeth#321	H/D exchange D_8/D_9
199.13413	10.26	$C_{11}H_{20}O_3$	199.13397	0.811	bemeth#25	co-eluting isomer, clearly differential in *endo*, D_{11}
215.12900	10.46	$C_{11}H_{20}O_4$	215.12888	0.559	bemeth#39	minor
201.14983	10.71	$C_{11}H_{22}O_3$	201.14962	1.050	bemeth#203	minor
591.17166	10.96	$C_{22}H_{33}N_4O_{13}P$	591.17090	1.297	gluric#423	MOGL, enriched *endo*. D_{12}
607.16623	10.98	$C_{22}H_{33}N_4O_{14}P$	607.16581	0.690	gluric#432	MOGL, enriched *endo*
256.15555	11.36	$C_{13}H_{23}NO_4$	256.15543	0.472	bemeth#53	minor
286.16616	11.37	$C_{14}H_{25}NO_5$	286.16600	0.574	bemeth#7	major, D_{12}
256.15555	11.42	$C_{13}H_{23}NO_4$	256.15543	0.472	bemeth#54	minor, D_{12}

286.16624	11.57	$C_{14}H_{25}NO_5$	286.16600	0.873	bemeth#72	secondary, D_{12}
256.15535	11.76	$C_{13}H_{23}NO_4$	256.15543	0.301	bemeth#5	major, D_{12}
256.15558	11.93	$C_{13}H_{23}NO_4$	256.15543	0.571	bemeth#52	minor
284.15051	11.95	$C_{14}H_{23}NO_5$	284.15035	0.568	bemeth#721	minor
268.15553	12.19	$C_{14}H_{23}NO_4$	268.15543	0.365	bemeth#621	minor
270.17116	12.46	$C_{14}H_{25}NO_4$	270.17108	0.275	bemeth#6	major, D_{12}
199.13412	12.59	$C_{11}H_{20}O_3$	199.13397	0.794	bemeth#23	unknown structure, D_{12},
201.14976	12.62	$C_{11}H_{22}O_3$	201.14962	0.718	bemeth#202	minor
270.17118	12.65	$C_{14}H_{25}NO_4$	270.17108	0.350	bemeth#62	secondary, D_{12}
199.13408	12.67	$C_{11}H_{20}O_3$	199.13397	0.580	bemeth#22	*2S,3S* (*anti*), minor, D_{12}
199.13408	12.83	$C_{11}H_{20}O_3$	199.13397	0.580	bemeth#2	*2R,3S* (*syn*), major, D_{12}
197.11838	12.89	$C_{11}H_{18}O_3$	197.11832	0.300	bemeth#221	
540.20039	13.18	$C_{25}H_{36}NO_{10}P$	540.20041	0.033	iglu#201	MOGL, enrichced *exo*, D_{12}
252.16065	13.20	$C_{14}H_{23}NO_3$	252.16052	0.523	bemeth#521	
201.14977	13.52	$C_{11}H_{22}O_3$	201.14962	0.762	bemeth#201	
199.13413	14.37	$C_{11}H_{20}O_3$	199.13397	0.807	bemeth#24	unknown structure
183.13928	26.65	$C_{11}H_{20}O_2$	183.13905	1.239	bemeth#1	Long HPLC method, post-column ion pairing
**ES(+) Obs. *m/z***	**RT (min)**	**Molecular Formula**	**ES(+) Theor. *m/z***	***m/z* error (ppm)**	**SMID-DB #**	**Comments**
260.18523	8.90	$C_{13}H_{25}NO_4$	260.18564	1.576	bemeth#101	
244.19040	11.56	$C_{13}H_{25}NO_3$	244.19072	1.330	bemeth#102	
246.20612	12.12	$C_{13}H_{27}NO_3$	246.20637	1.022	bemeth#103	enriched in *hacl-1(tm6725)*
246.20606	12.36	$C_{13}H_{27}NO_3$	246.20637	1.272	bemeth#104	detected only in *hacl-1(tm6725)*
230.21103	12.98	$C_{13}H_{27}NO_2$	230.21146	1.689	bemeth#105	

Supplementary Table 3. Primers used for genotyping.

Strain	Primer Sequence
FCS7 *hacl-1(tm6725)* II	Fwd: GAAGTAGGAATGGCAGCACAAG
	Rev: GGCACTGCTGAACTTGTGTAGCTC
	Int. Fwd: CTGCTGGCCTGTAGTCTGTATTG
FCS40 *fcmt-1(gk155709)* II	Fwd: CCGAGCACTCTGGGAGATTG
	Rev: GTGCTCACCAAATCCCACCG
FCS20 *fcmt-1(tm2382)* II	Fwd: CATCCAGGCGCTGGAATTC
	Rev: GCTCAATCGAAACCCGTGC

Supplementary Table 4. Primers used for gene expression analysis by RT-PCR.

Gene	Primer Sequence
act-1	Fwd: ACGACGAGTCCGGCCCATCC
	Rev: GAAAGCTGGTGGTGACGATGGTT
fat-7	Fwd: GGAAGGAGACAGCATTCATTGCG
	Rev: GTCTTGTGGGAATGTGTGGTGG
fat-6	Fwd: GGAAATTGTGTGGCGTAACG
	Rev: GTATGATTTGTGGGACCAGAGACG

Supplementary Table 5. ^{1}H NMR spectroscopic data of natural becyp#2, methanol-d_4 (800 MHz, CD_3OD).

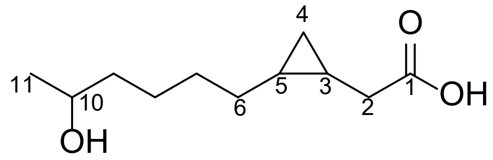

becyp#2

Position	Proton	^{1}H chemical shift [ppm]	[^{1}H,^{1}H]-Coupling constants [Hz]
1			
2	2-H_a<br>2-H_b	2.16<br>2.28	$J_{2\text{-Ha},2\text{-Hb}}$ = 15.8, $J_{2\text{-Ha},2\text{-}3}$ = 7.8,<br>$J_{2\text{-Hb},3}$ = 6.0,
3	3-H	1.09	
4	4-H_a<br>4-H_b	-0.15<br>0.68	
5	5-H	0.78	
6-9	6-9H	1.3-1.5	
10	10-H	3.72	$J_{9,10} \approx J_{10,11}$ = 6.1
11	11-H	1.14	$J_{10,11}$ = 6.1

Supplementary References

1. Wilson, S.R., and Prodan, K.A. (1976). The synthesis and stereochemistry of cascarillic acid. Tetrahedron Lett. *17*, 4231–4234.
2. Naef, R., Velluz, A., and Jaquier, A. (2007). New Volatile Sulfur-Containing Constituents in a Simultaneous Distillation−Extraction Extract of Red Bell Peppers (Capsicum annuum). J. Agric. Food Chem. *56*, 517–527.
3. Gao, Y., Sharpless, K.B., Klunder, J.M., Hanson, R.M., Ko, S.Y., and Masamune, H. (1987). Catalytic Asymmetric Epoxidation and Kinetic Resolution: Modified Procedures Including in Situ Derivatization. J. Am. Chem. Soc. *109*, 5765–5780.
4. Francke, W., Schröder, F., Philipp, P., Meyer, H., Sinnwell, V., and Gries, G. (1996). Identification and synthesis of new bicyclic acetals from the mountain pine beetle, Dendroctonus ponderosae hopkins (Col.: Scol.). Bioorg. Med. Chem. *4*, 363–374.
5. Paquette, L.A., Eizember, R.F., Chapman, O.L., Rettig, T.A., Griswold, A.A., Dutton, A.I., Fitton, P., Abrahamson, W., F Littler, J.G., Vo, K., et al. (1963). Photochemistry of the cis- and trans-Bicyclo [6.1.0]nonan-2-ones1 Wiberg and deMeijere have estimated that an enthalpy difference of 3.5 kcal/mole separates 1 from 2,4 (6) E. Tetrahedron Lett *21*, 59.
6. Fallis, A.G., and Souweha, M.S. (2008). Sigmatropic Rearrangements. Polyynes, Arynes, Enynes, and Alkynes *53*, 1.
7. Coggins, A.J., and Powner, M.W. (2016). Prebiotic synthesis of phosphoenol pyruvate by α-phosphorylation-controlled triose glycolysis. Nat. Chem. 2016 94 *9*, 310–317.
8. Davis, F.A., Haque, M.S., Ulatowski, T.G., and Towson, J.C. (1986). Asymmetric Oxidation of Ester and Amide Enolates Using New (Camphorylsulfonyl)oxaziridines. J. Org. Chem. *51*, 2402–2404.
9. Hoye, T.R., Jeffrey, C.S., and Shao, F. (2007). Mosher ester analysis for the determination of absolute configuration of stereogenic (chiral) carbinol carbons. Nat. Protoc. 2007 210 *2*, 2451–2458.

NMR Spectra of Synthetic Compounds

cis-3,4-methylenedecanoic acid, becyp#1, ^{1}H NMR spectrum (500 MHz, $CDCl_3$)

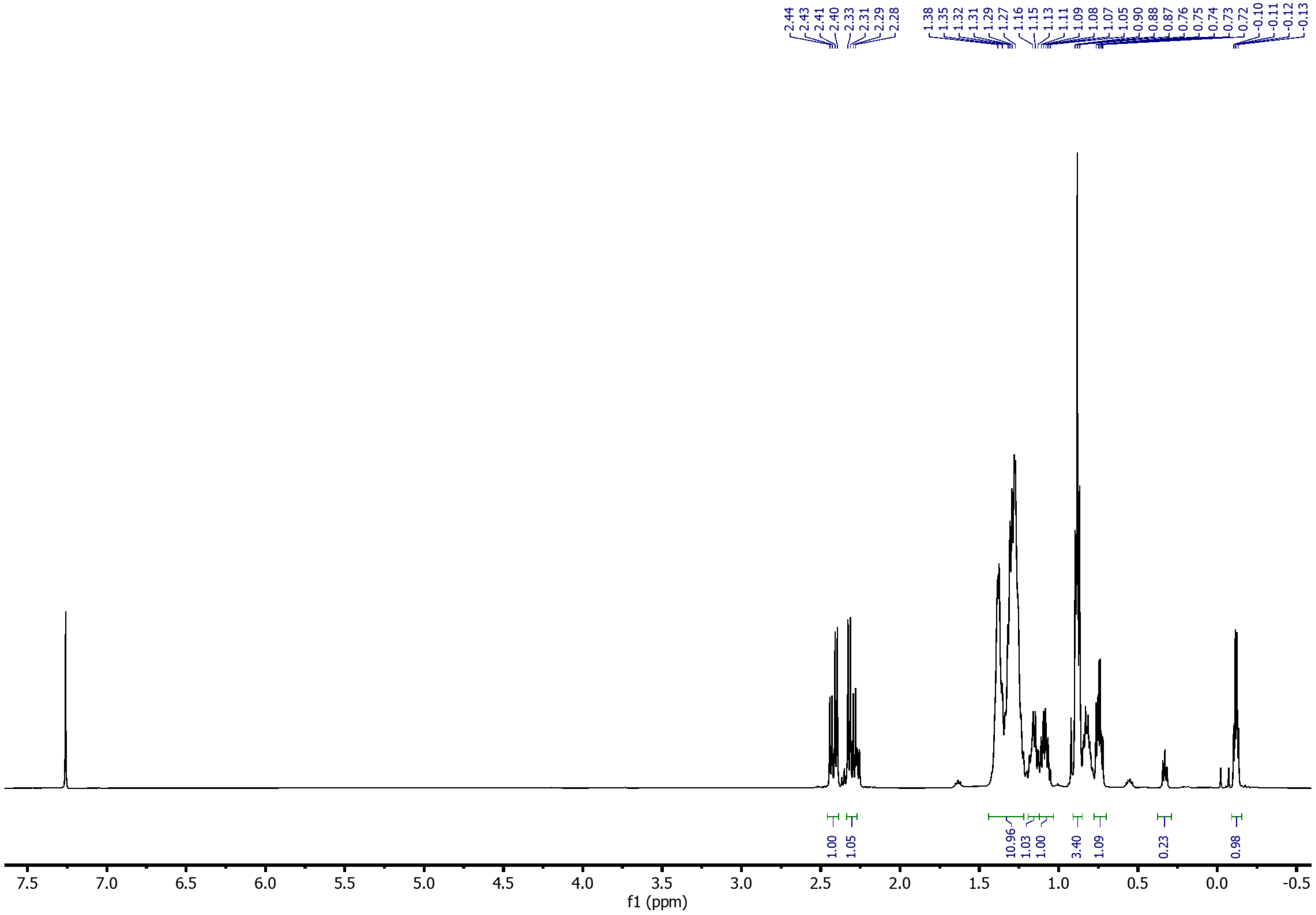

cis-3,4-methylenedecanoic acid, becyp#1, ^{13}C NMR spectrum (125 MHz, $CDCl_3$)

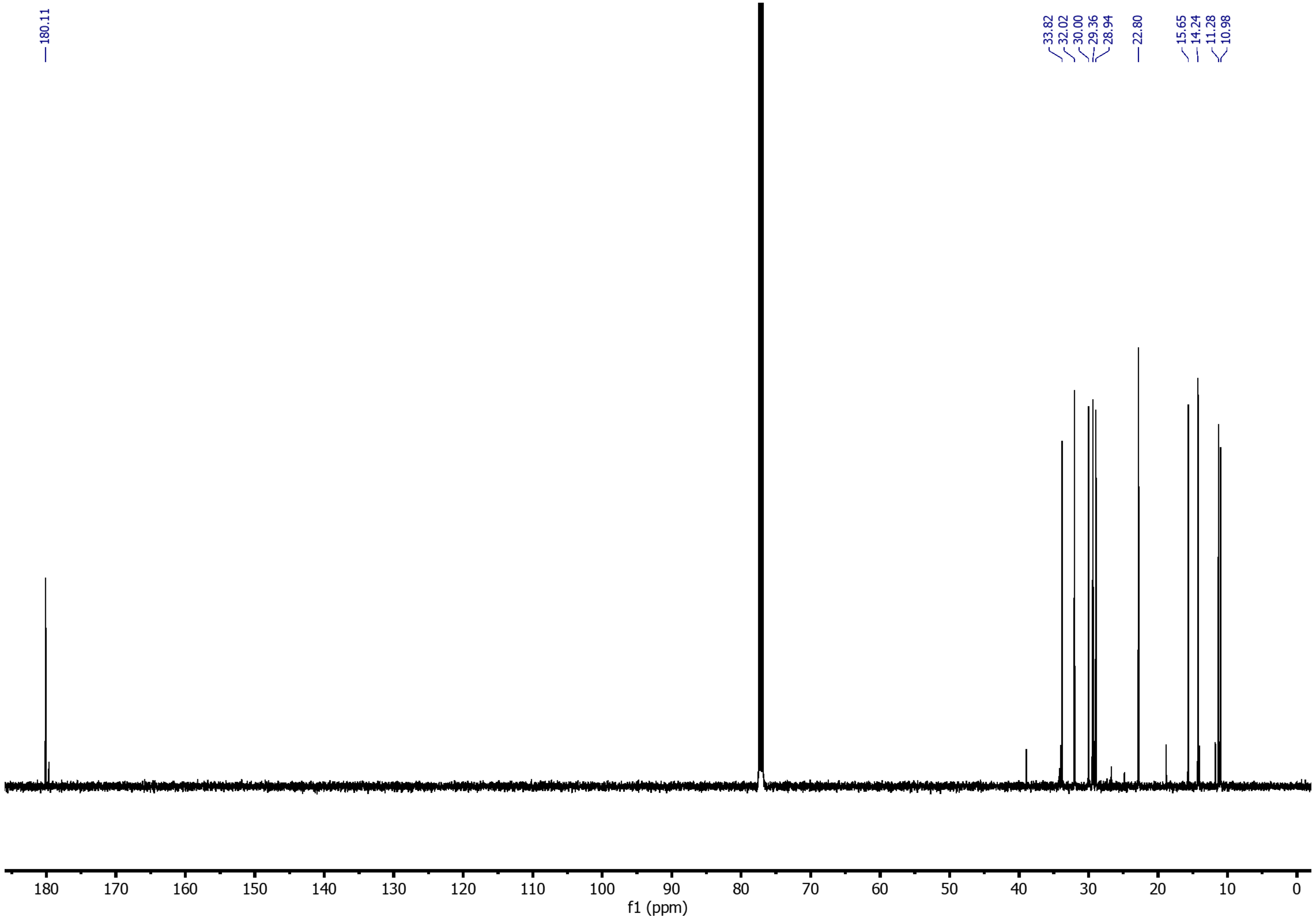

(*E*)-4-Hydroxy-non-2-ene (**5**), ^{1}H NMR spectrum (500 MHz, $CDCl_3$)

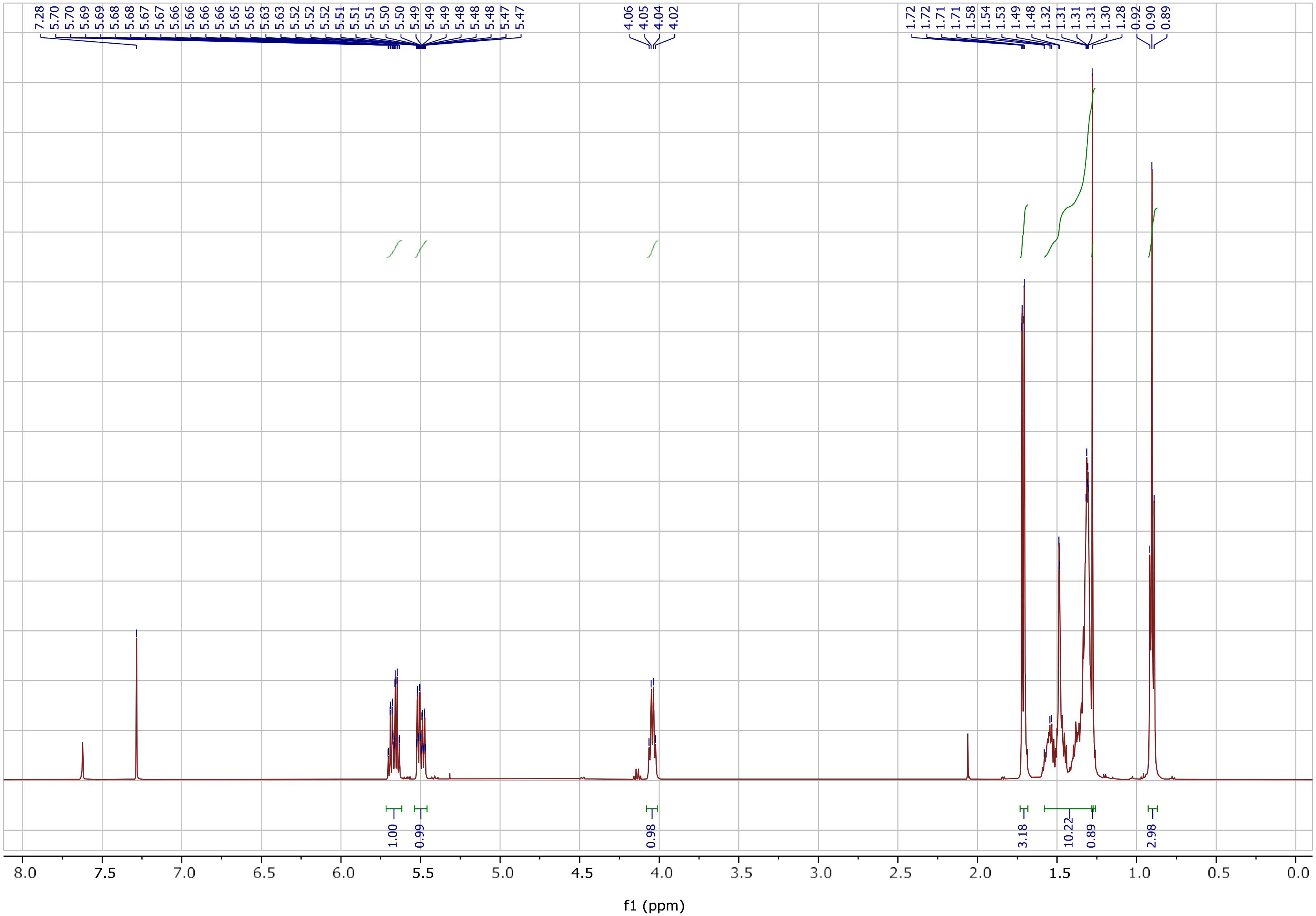

(E)-4-Hydroxy-non-2-ene (**5**), ^{13}C NMR spectrum (125 MHz, $CDCl_3$)

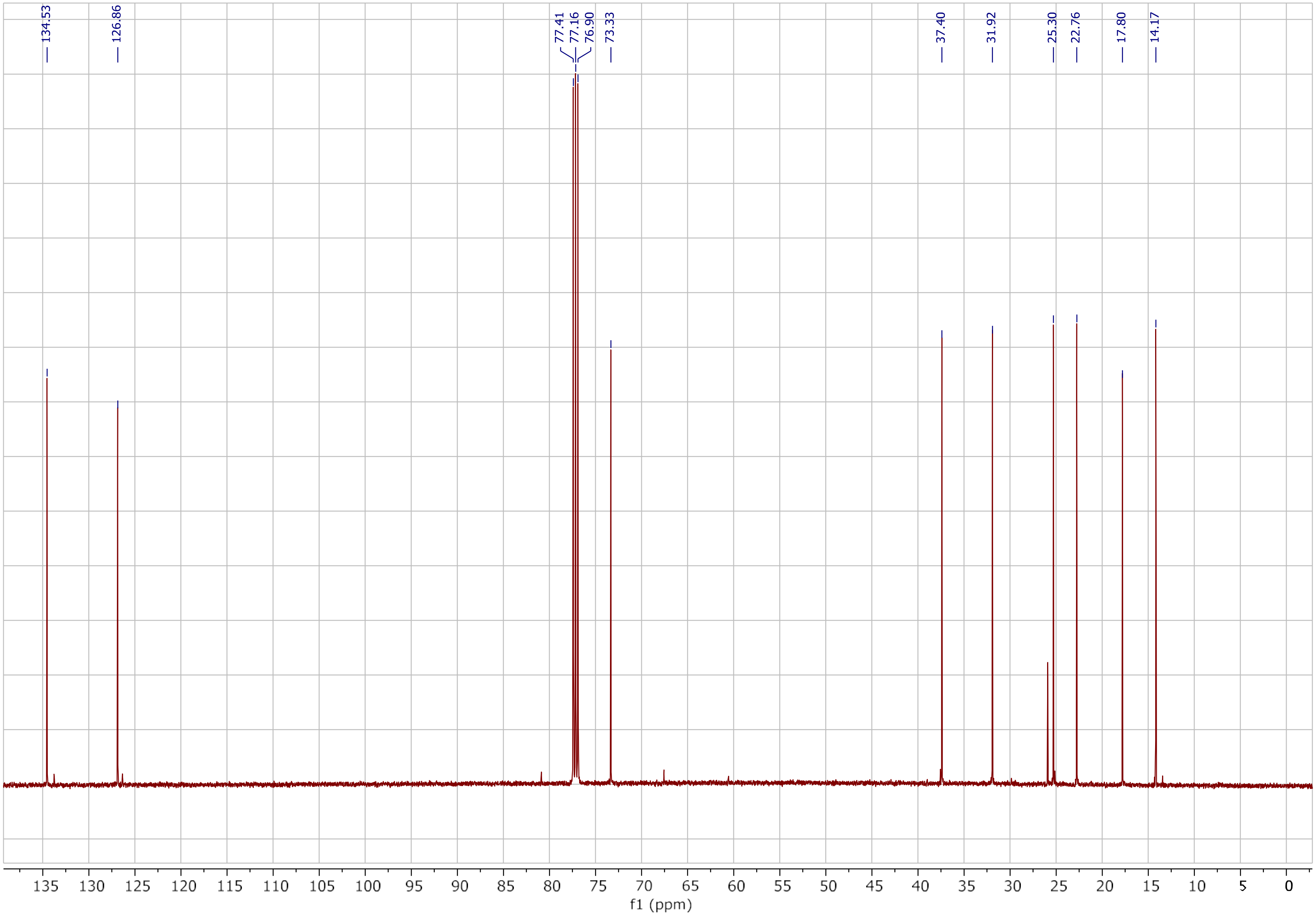

(S,E)-4-(Vinyloxy)-non-2-ene (**6**), ^{1}H NMR spectrum(500 MHz, $CDCl_3$)

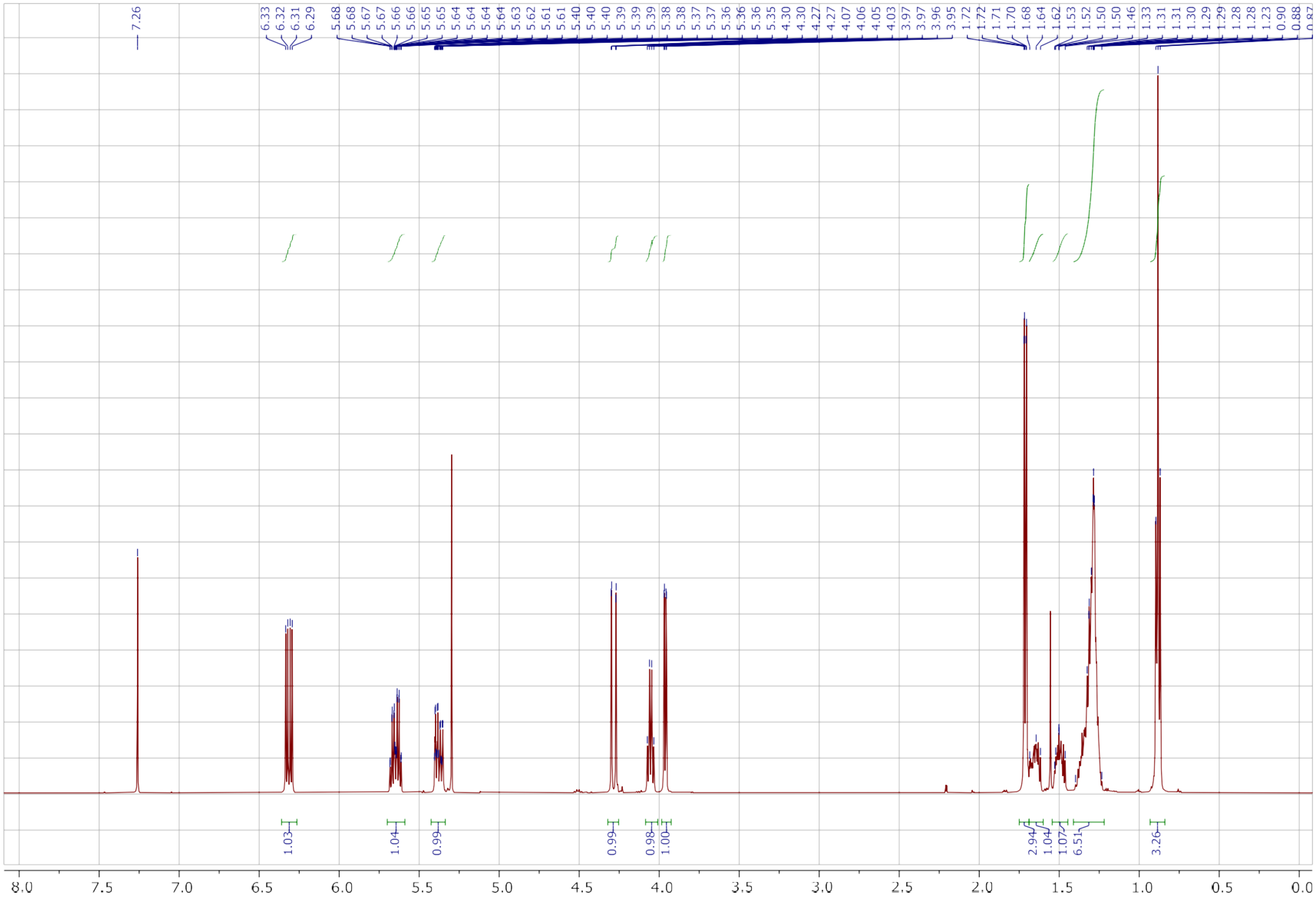

(*S,E*)-4-(Vinyloxy)-non-2-ene (**6**), ^{13}C NMR spectrum (125 MHz, $CDCl_3$)

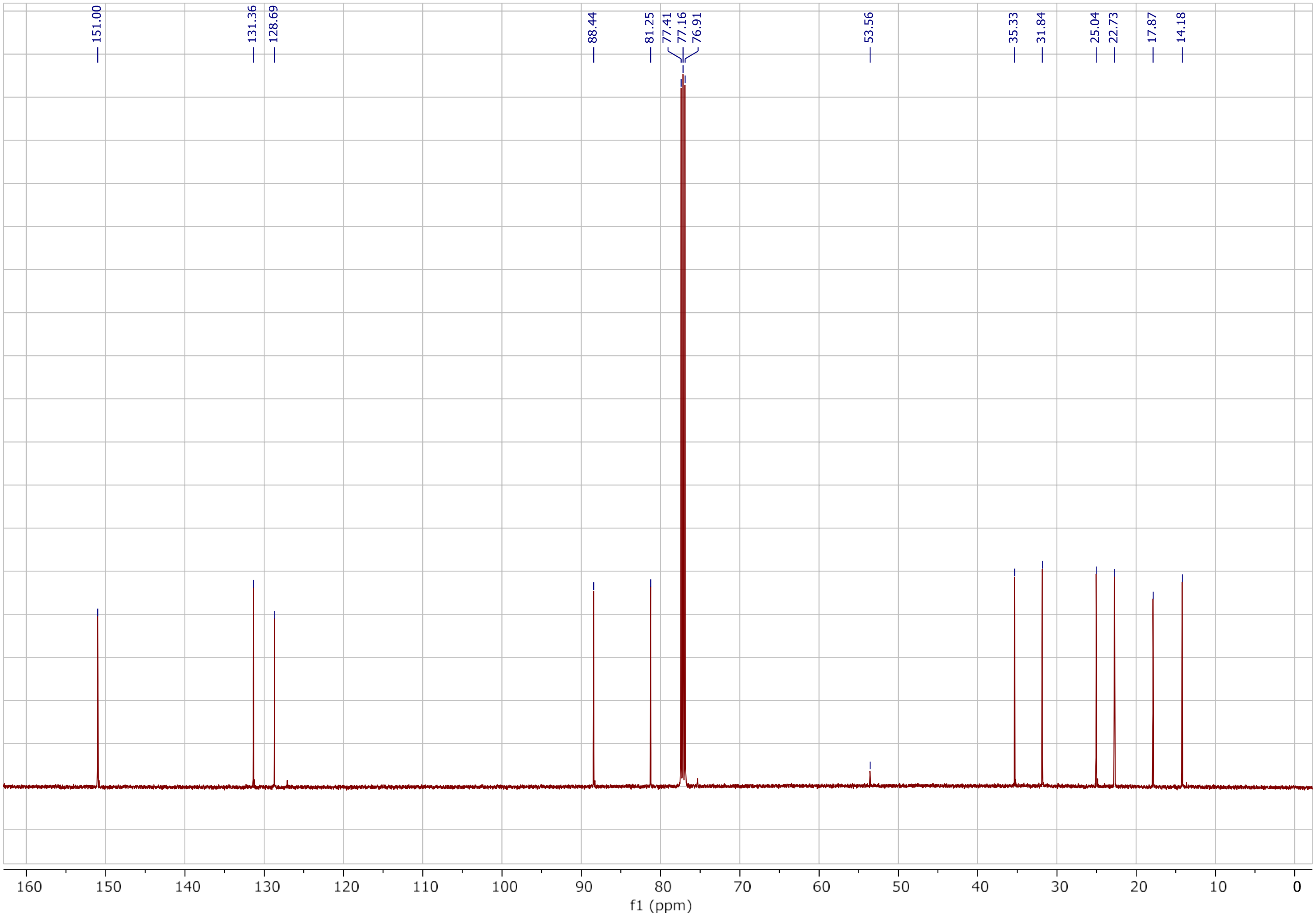

(*R,E*)-3-Methyl-dec-4-enal (**7**), ^{1}H NMR spectrum (500 MHz, $CDCl_3$)

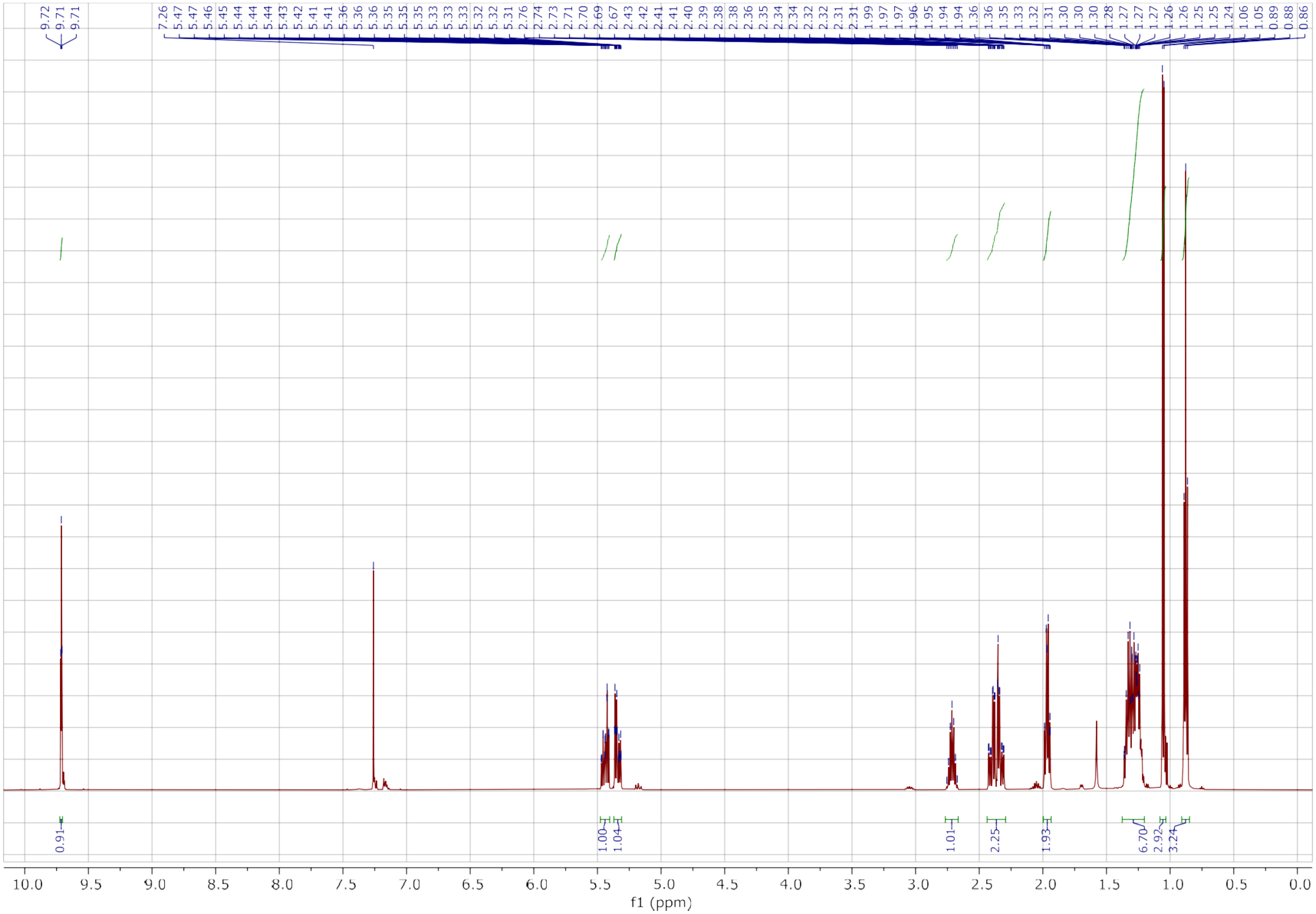

(*R,E*)-3-Methyl-dec-4-enal (**7**), ^{13}C NMR spectrum (125 MHz, $CDCl_3$)

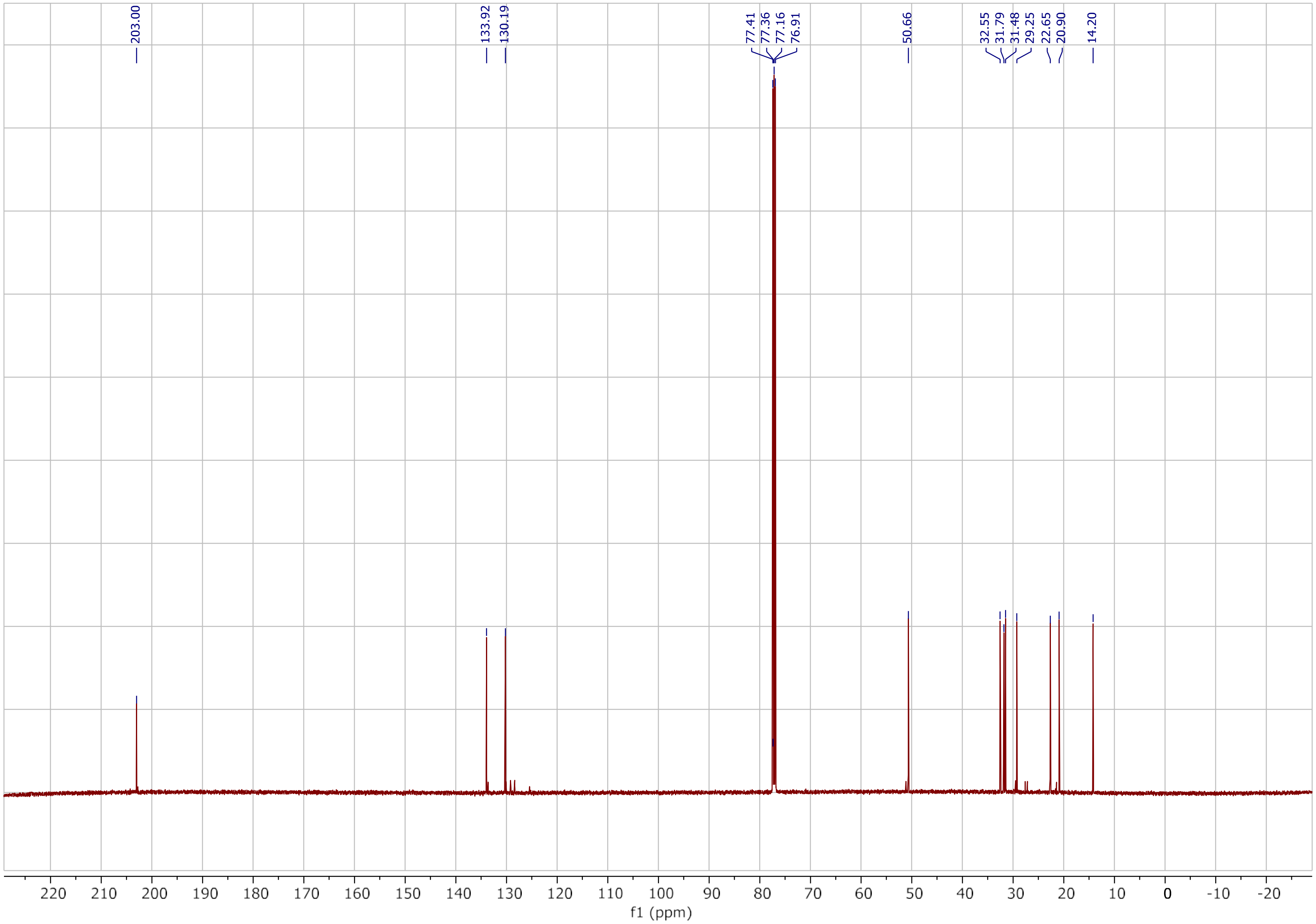

((*R,E*)-3-Methyl-dec-4-enoic acid, (*R*)-bemeth#1, ^{1}H NMR spectrum (500 MHz, $CDCl_3$)

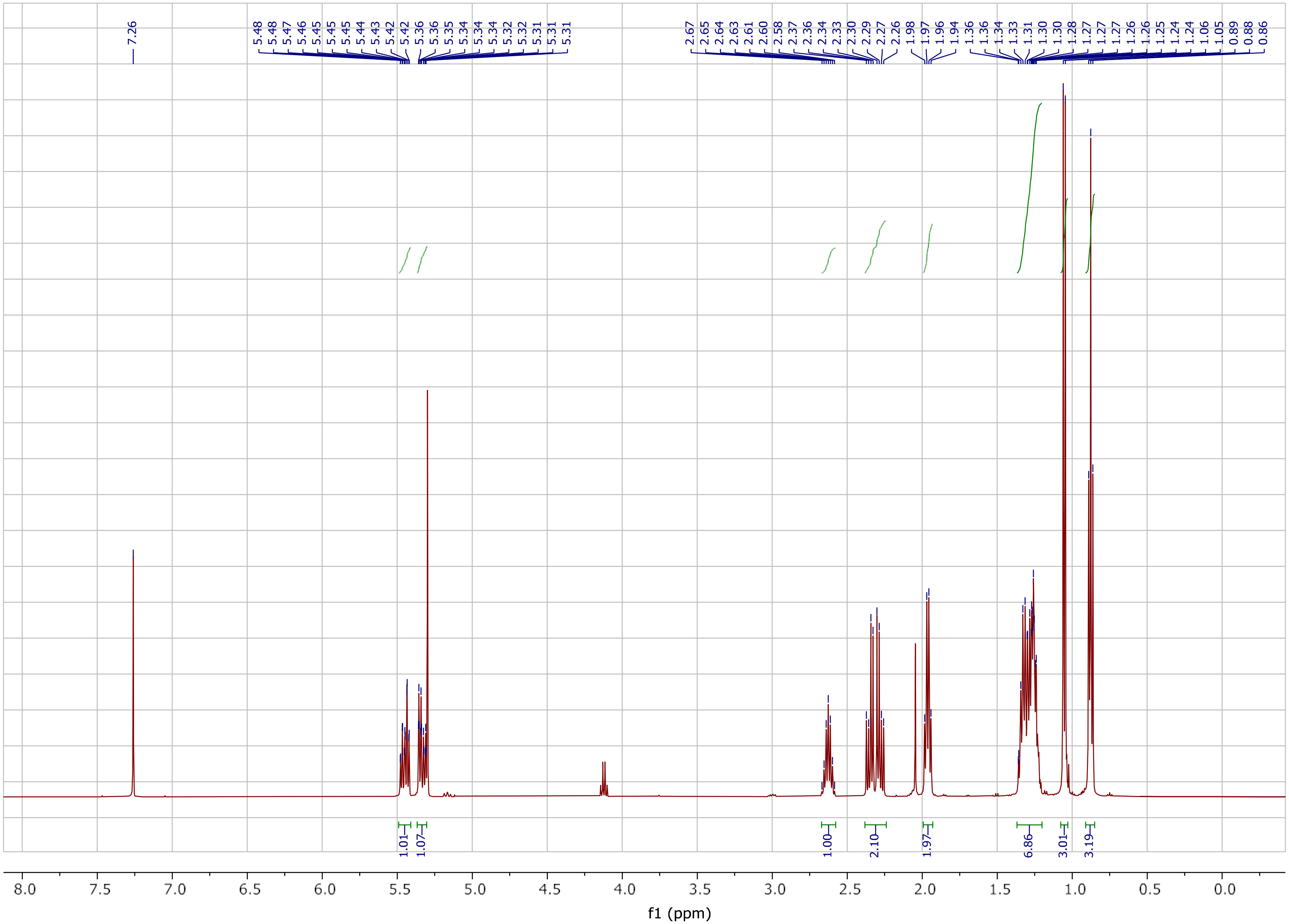

(*R,E*)-3-Methyl-dec-4-enoic acid, (*R*)-bemeth#1, ^{13}C NMR spectrum(125 MHz, $CDCl_3$)

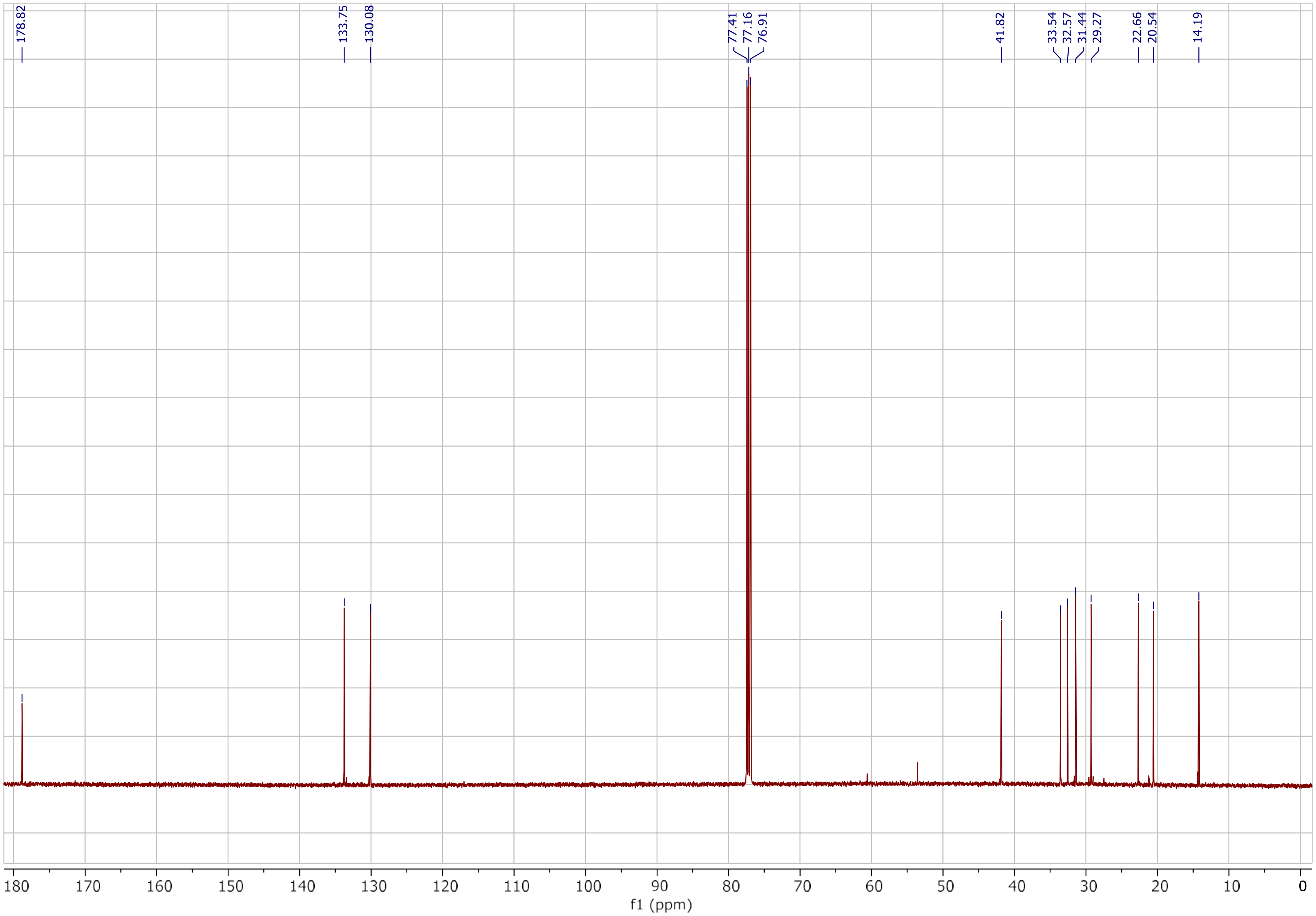

Methyl-*(R,E)*-3-methyl-dec-4-enoate (**8**), ^{1}H NMR spectrum (500 MHz, $CDCl_3$)

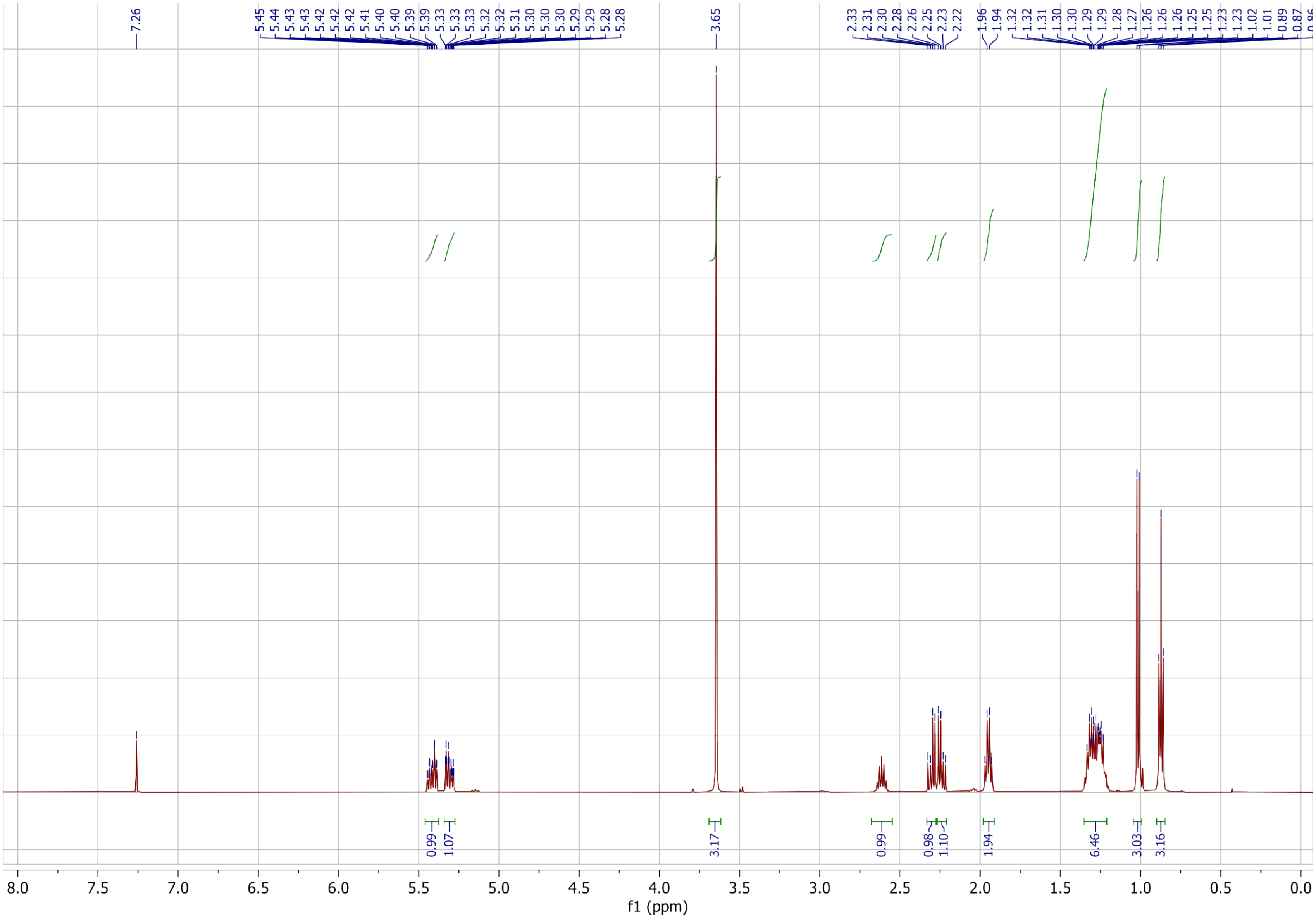

Methyl-*(R,E)*-3-methyl-dec-4-enoate (**8**), ^{13}C NMR spectrum (125 MHz, $CDCl_3$)

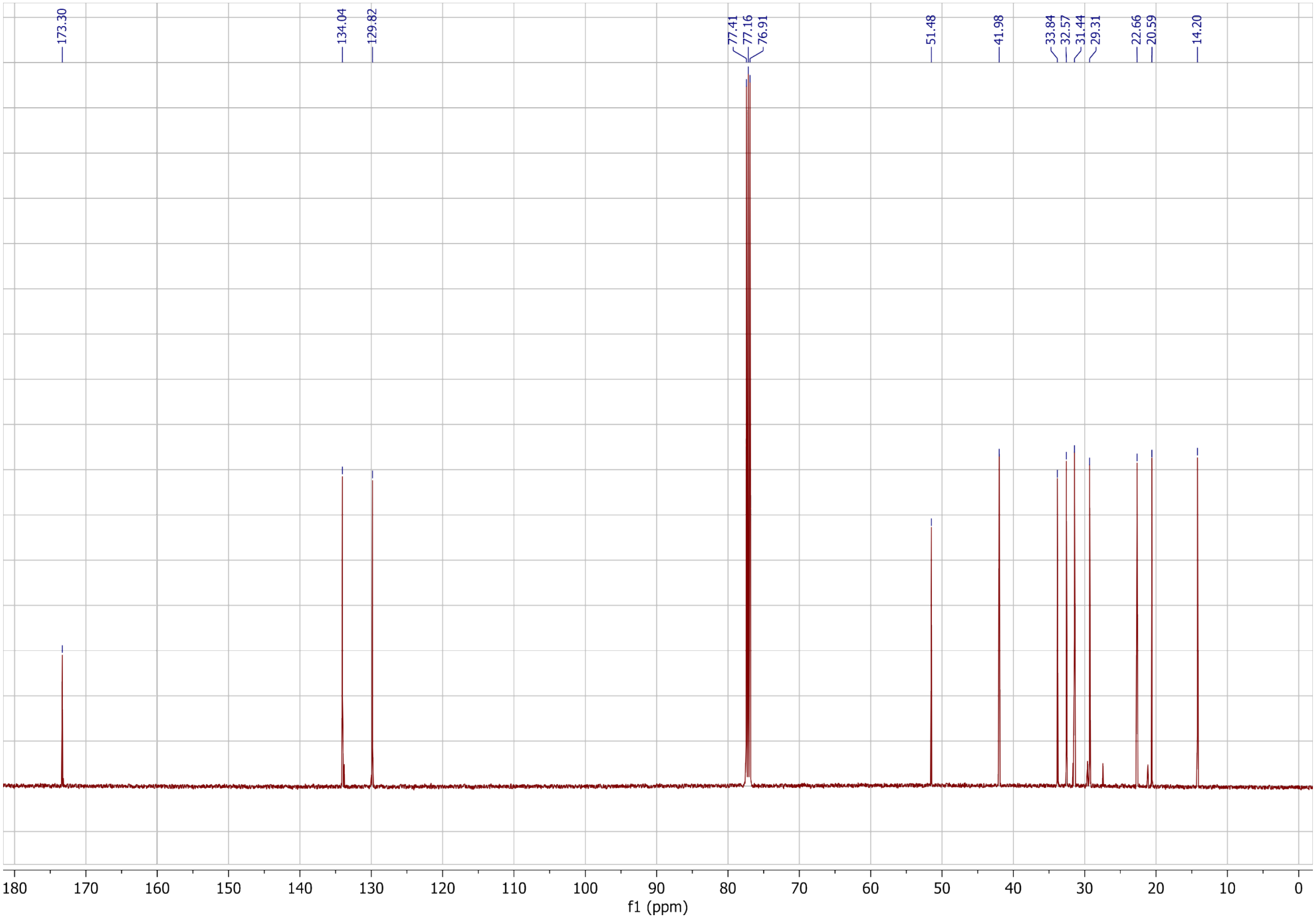

Methyl-*(2R,3S,E)*-2-hydroxy-3-methyl-dec-4-enoate (**9**), ^{1}H NMR spectrum (500 MHz, $CDCl_3$)

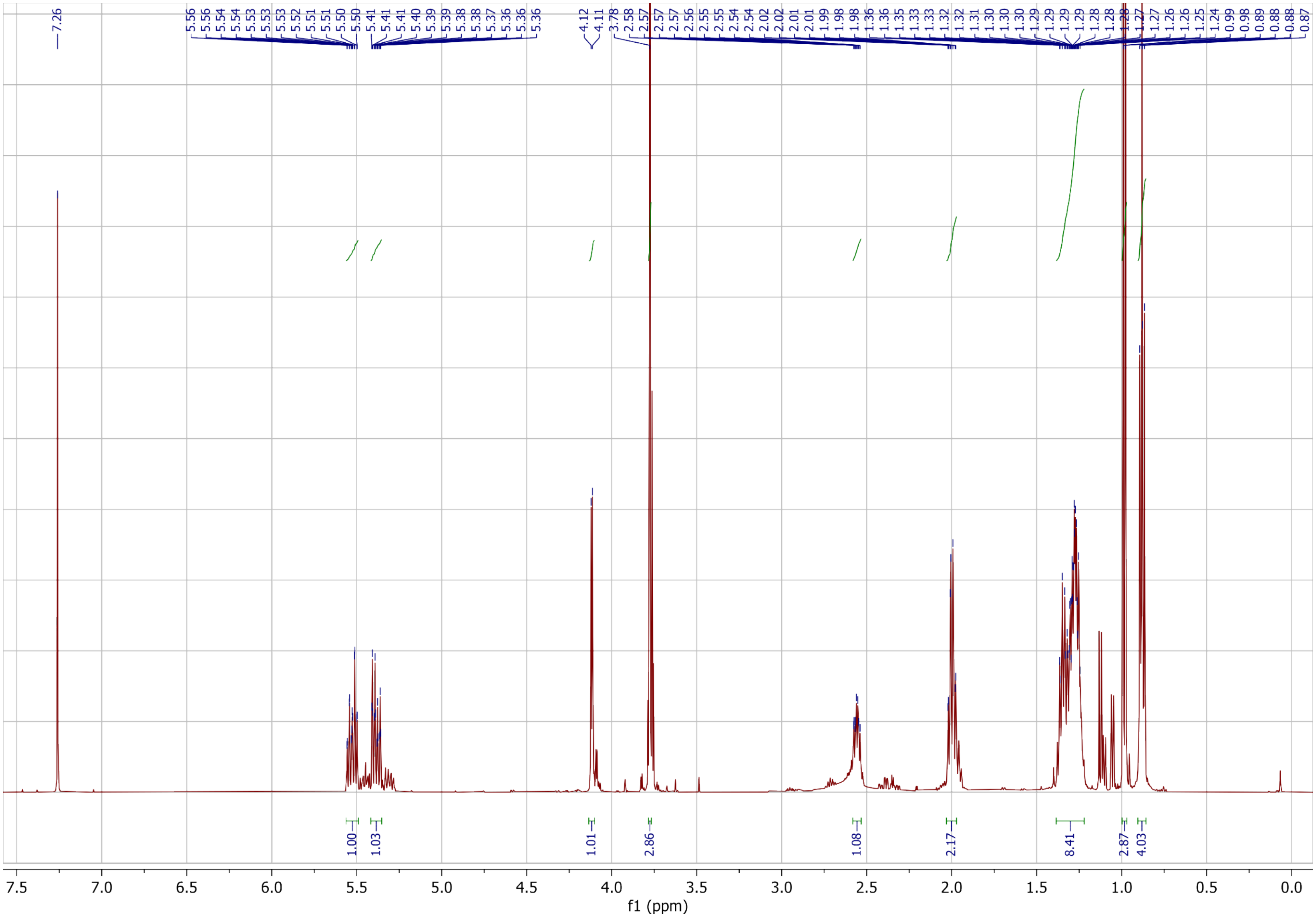

Methyl-*(2R,3S,E)*-2-hydroxy-3-methyl-dec-4-enoate (**9**) , ^{13}C NMR spectrum (125 MHz, $CDCl_3$)

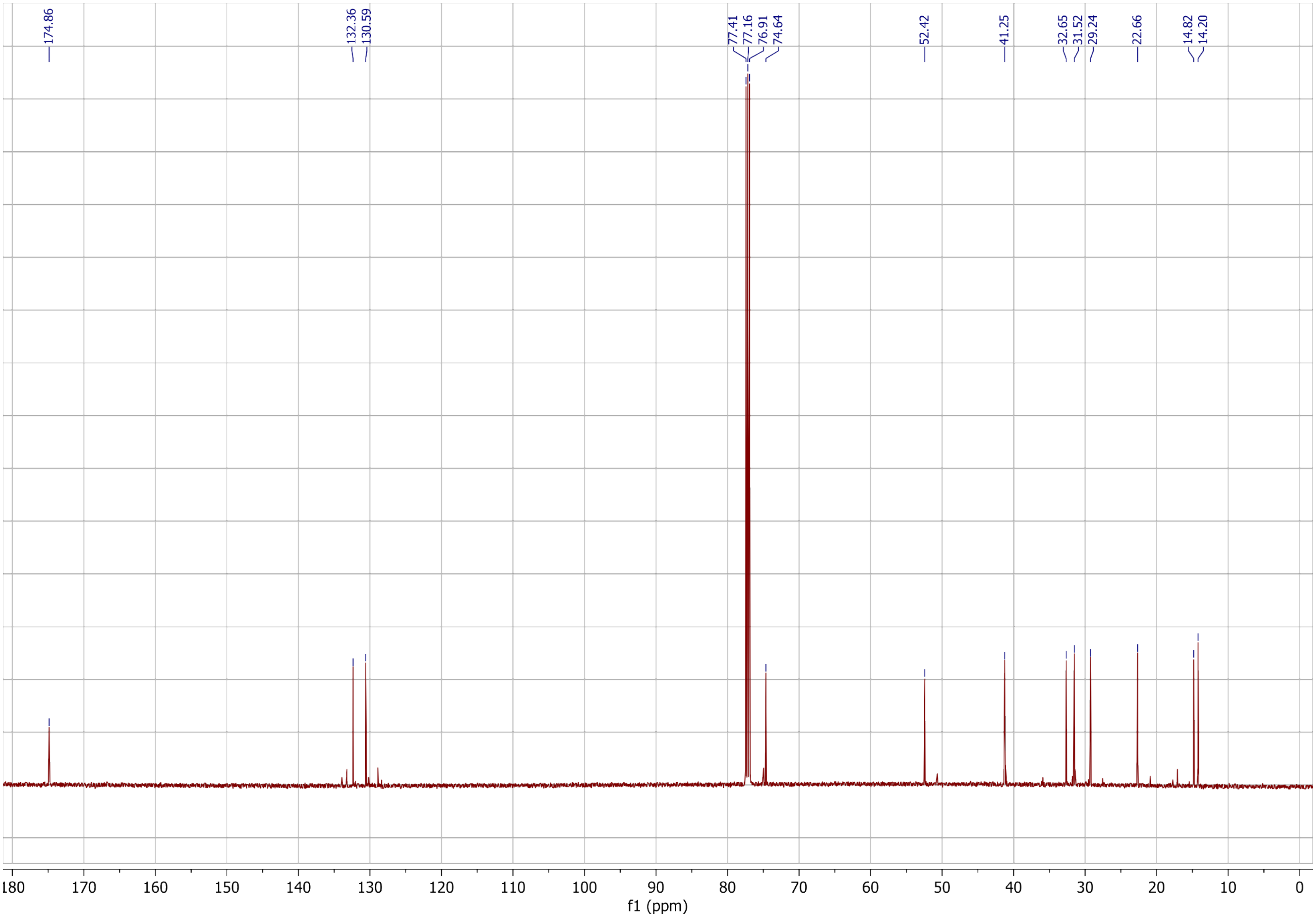

(2R,3S,E)-2-hydroxy-3-methyl-dec-4-enoic acid, (2R,3S)-bemeth#2, ^{1}H NMR spectrum (500 MHz, $CDCl_3$)

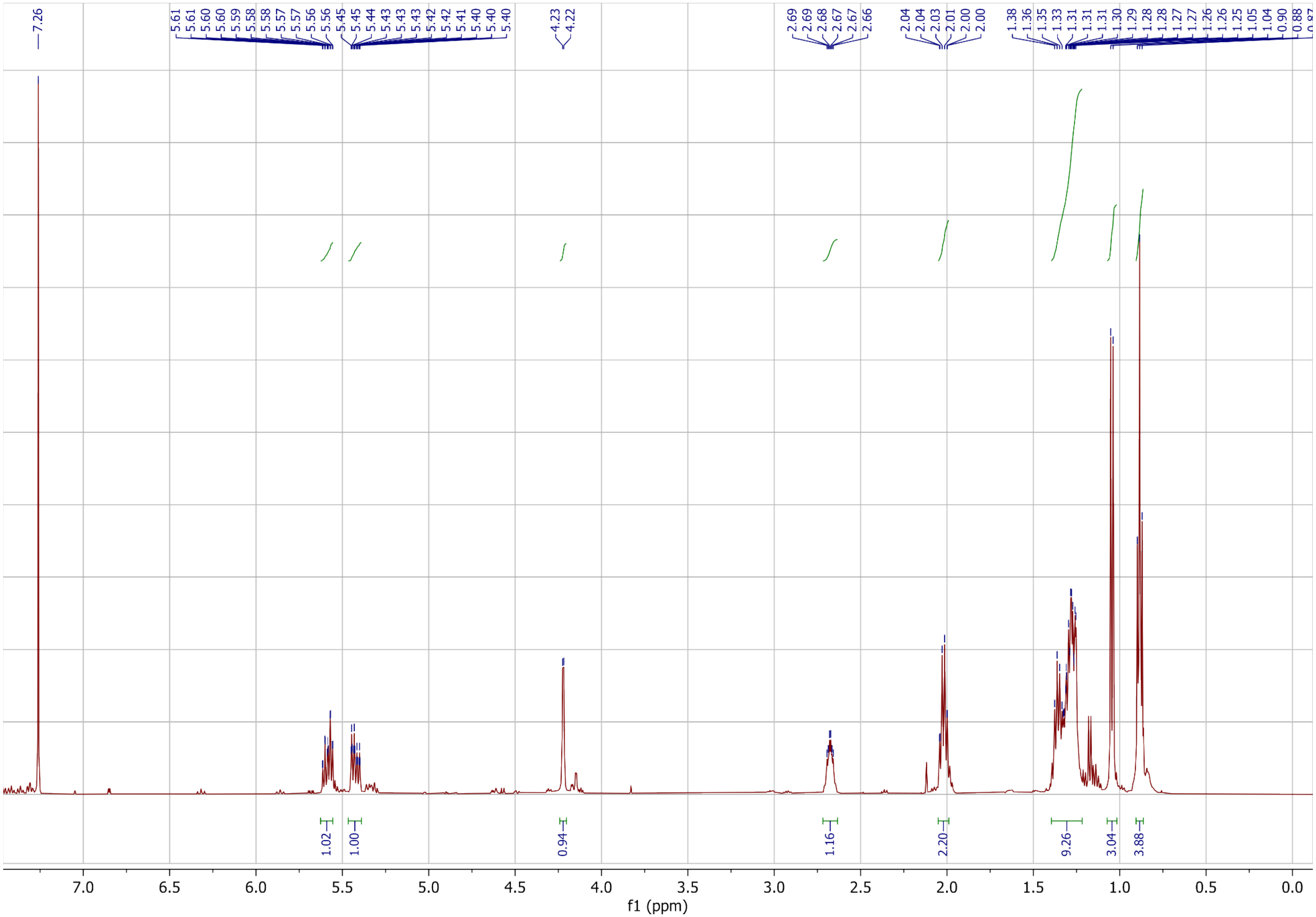

(2R,3S,E)-2-hydroxy-3-methyl-dec-4-enoic acid, (*2R,3S*)-bemeth#2, ^{13}C NMR spectrum (125 MHz, $CDCl_3$)

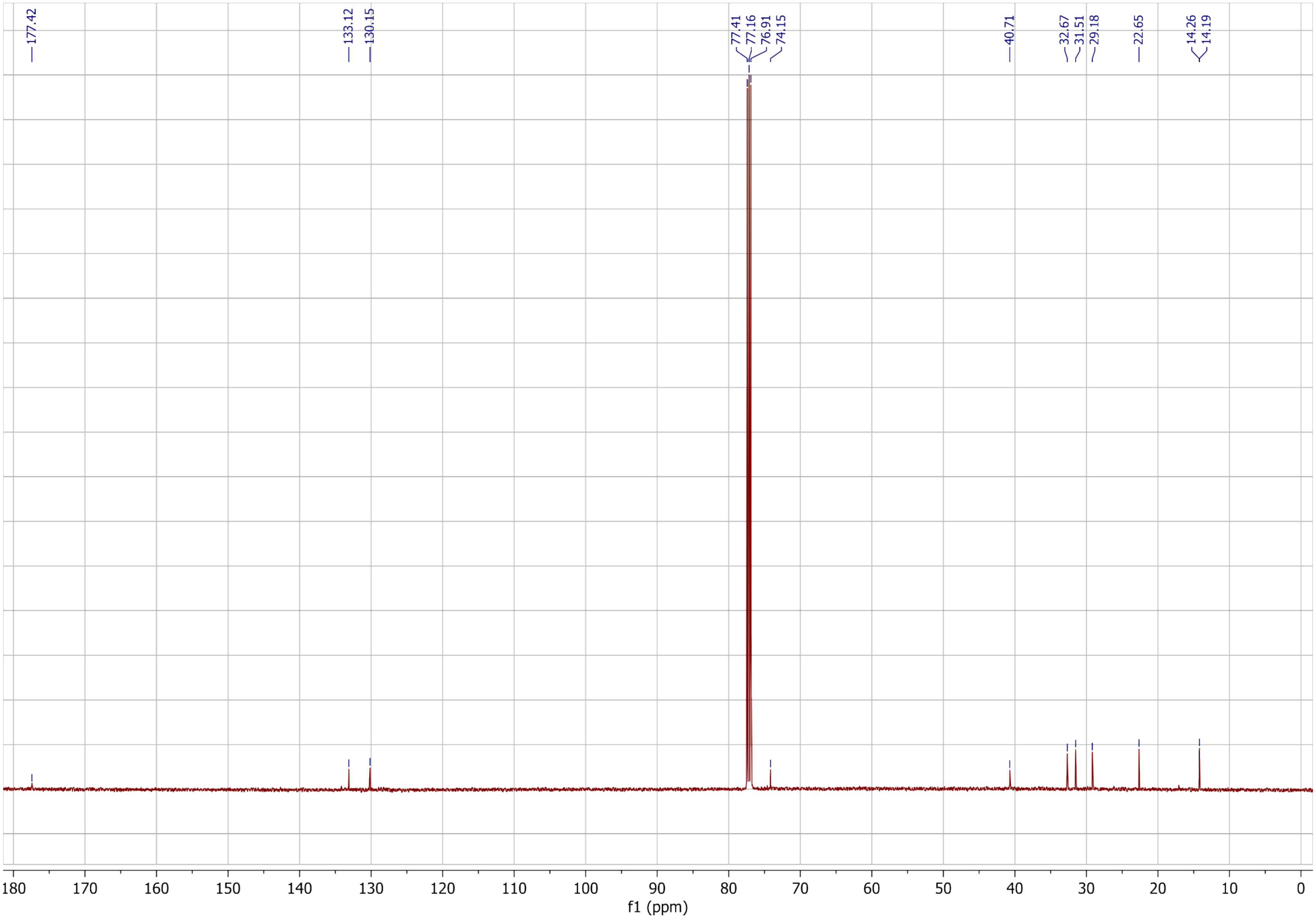

Comparison of ^{1}H NMR (400 MHz, CD_3OD) spectra for Mosher analysis. Earlier and later eluting diastereomers were separated following derivatization with (*R*)-MTPA-Cl. Derivatization of the major natural isomer, (*2R,3S*)-bemeth#2, exhibited identical chromatographic retention and chemical shifts as the later eluting fraction.

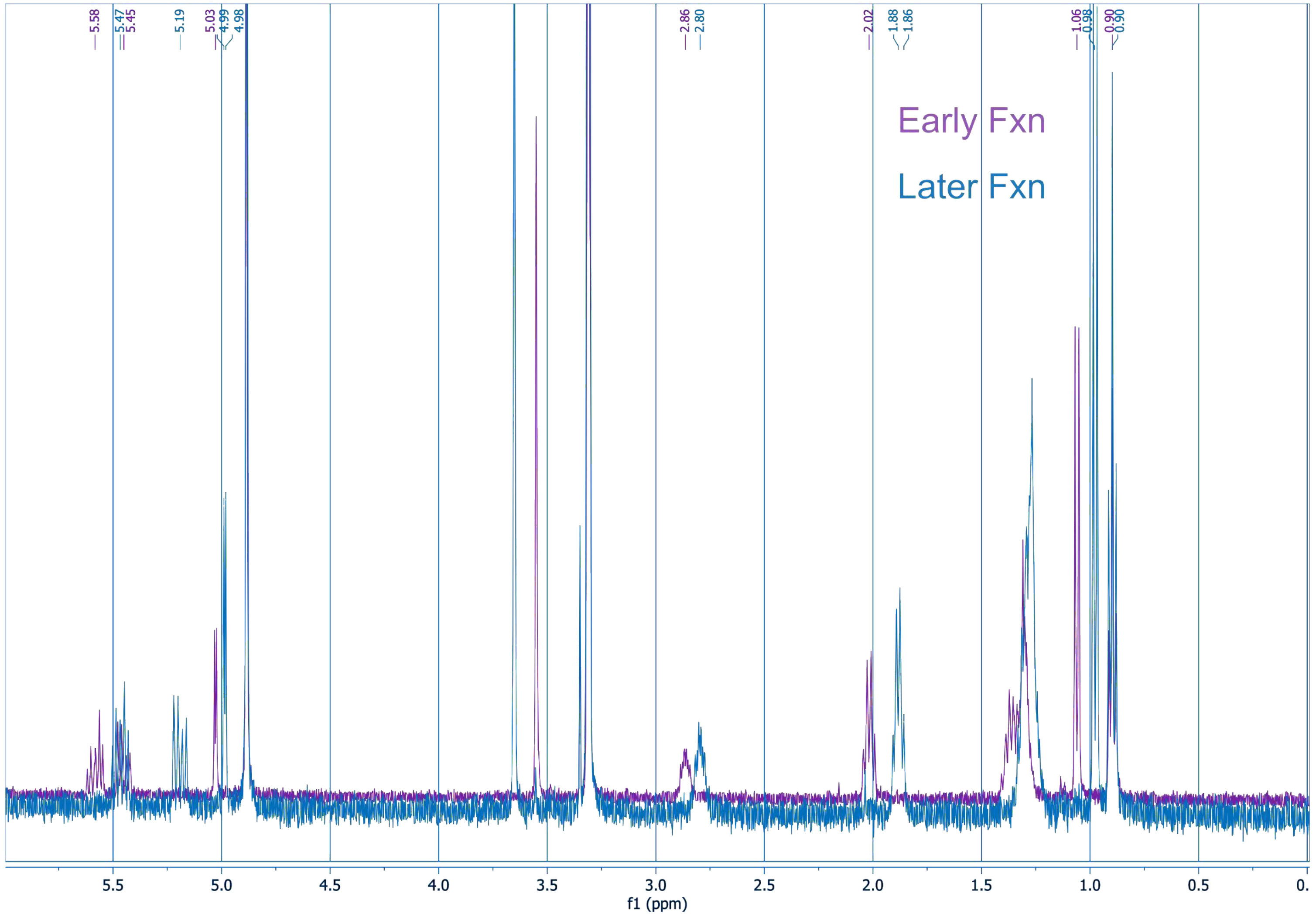

Supplement: Supplementary file 1 — Supplementary Information [file 41467_2024_45782_MOESM1_ESM.pdf]
