# Supplementary material for: Evolutionarily related host and microbial pathways regulate fat desaturation in C. elegans
Source: Nat Commun. 2024 Feb 19;15:1520. doi: 10.1038/s41467-024-45782-2 (PMC10876521; doi:10.1038/s41467-024-45782-2)
Supplement: Supplementary file 3 — Reporting Summary [file 41467_2024_45782_MOESM3_ESM.pdf]

nature portfolio

Corresponding author(s): Frank Schroeder

Last updated by author(s): 1/26/2024

Reporting Summary

Nature Portfolio wishes to improve the reproducibility of the work that we publish. This form provides structure for consistency and transparency in reporting. For further information on Nature Portfolio policies, see our Editorial Policies and the Editorial Policy Checklist.

Statistics

For all statistical analyses, confirm that the following items are present in the figure legend, table legend, main text, or Methods section.

n/a	Confirmed	
☐	☒	The exact sample size (*n*) for each experimental group/condition, given as a discrete number and unit of measurement
☐	☒	A statement on whether measurements were taken from distinct samples or whether the same sample was measured repeatedly
☐	☒	The statistical test(s) used AND whether they are one- or two-sided<br>*Only common tests should be described solely by name; describe more complex techniques in the Methods section.*
☒	☐	A description of all covariates tested
☐	☒	A description of any assumptions or corrections, such as tests of normality and adjustment for multiple comparisons
☐	☒	A full description of the statistical parameters including central tendency (e.g. means) or other basic estimates (e.g. regression coefficient) AND variation (e.g. standard deviation) or associated estimates of uncertainty (e.g. confidence intervals)
☐	☒	For null hypothesis testing, the test statistic (e.g. *F*, *t*, *r*) with confidence intervals, effect sizes, degrees of freedom and *P* value noted<br>*Give P values as exact values whenever suitable.*
☒	☐	For Bayesian analysis, information on the choice of priors and Markov chain Monte Carlo settings
☒	☐	For hierarchical and complex designs, identification of the appropriate level for tests and full reporting of outcomes
☒	☐	Estimates of effect sizes (e.g. Cohen's *d*, Pearson's *r*), indicating how they were calculated

Our web collection on statistics for biologists contains articles on many of the points above.

Software and code

Policy information about availability of computer code

Data collection	MS data acquisition was performed using a Vanquish HPLC system controlled by Chromeleon Software (ThermoFisher Scientific v7.3) and coupled to an Orbitrap Q-Exactive HF mass spectrometer controlled by Xcalibur software (ThermoFisher Scientific v4.1.31.9), or by a Dionex Ultimate 3000 HPLC system coupled to an Oribtrap Q-Exactive mass spectrometer controlled by the same software. Protein sequence data was downloaded from the National Center for Biotechnology Information (NCBI) non-redundant (nr) database. Microscopy data acquisition was performed using a Leica M205FA stereomicroscope outfitted with a DFC7000T camera controlled by Leica Application Suite X software (v3.6.0.20104 Leica Microsystems). NMR spectra were recorded on Varian INOVA 600 (600 MHz) or Bruker AV 500 (500 MHz) spectrometers. RT-PCR was performed with SYBR green dye (Thermo Fisher Scientific cat. no 4367659) using a Bio-Rad C1000TM Thermal Cycler (Bio-Rad Laboratories Inc.)
Data analysis	RAW HPLC-HRMS data files were converted to mzXML file format using MSConvert (v3.0, ProteoWizard). Data was analyzed using Metaboseek software (current v0.9.9) available here: [https://doi.org/10.5281/zenodo.5725575] and Xcalibur Qual Browser (Thermo Scientific v4.1.31.9). Protein sequences were analyzed by MUSCLE alignment using MEGA 11 software [https://www.megasoftware.net/]. Quantification of micrographs was performed using ImageJ software (v1.54c). NMR spectra were processed and baseline corrected using MestreLabs MNOVA software (v11.0.0-17609). Statistical analysis was performed with Metaboseek software (v0.9.9.0), Microsoft Excel (v2302 Build 16.0.16130.20332), and with GraphPad Prism (v9.5.0.730).

For manuscripts utilizing custom algorithms or software that are central to the research but not yet described in published literature, software must be made available to editors and reviewers. We strongly encourage code deposition in a community repository (e.g. GitHub). See the Nature Portfolio guidelines for submitting code & software for further information.

Data

Policy information about availability of data

All manuscripts must include a data availability statement. This statement should provide the following information, where applicable:

- Accession codes, unique identifiers, or web links for publicly available datasets
- A description of any restrictions on data availability
- For clinical datasets or third party data, please ensure that the statement adheres to our policy

The HPLC-HRMS data generated during this study have been deposited in the MassIVE database under accession code MSV000092700 [doi:10.25345/C50R9MF18]. Detailed information about newly described C. elegans compounds can be accessed via the Small Molecule Identifier Database (SMID-DB, https://www.smid-db.org/). Protein sequence data was downloaded from the National Center for Biotechnology Information (NCBI) non-redundant (nr) database (https://www.ncbi.nlm.nih.gov/refseq/about/nonredundantproteins/)

Research involving human participants, their data, or biological material

Policy information about studies with human participants or human data. See also policy information about sex, gender (identity/presentation), and sexual orientation and race, ethnicity and racism.

Reporting on sex and gender	N/A
Reporting on race, ethnicity, or other socially relevant groupings	N/A
Population characteristics	N/A
Recruitment	N/A
Ethics oversight	N/A

Note that full information on the approval of the study protocol must also be provided in the manuscript.

Field-specific reporting

Please select the one below that is the best fit for your research. If you are not sure, read the appropriate sections before making your selection.

☒ Life sciences ☐ Behavioural & social sciences ☐ Ecological, evolutionary & environmental sciences

For a reference copy of the document with all sections, see nature.com/documents/nr-reporting-summary-flat.pdf

Life sciences study design

All studies must disclose on these points even when the disclosure is negative.

Sample size	No sample size calculations were performed. The sample sizes in the study were chosen based on prior experience, reasonable replication, and standards in the field (see, for example, Ma et al. 2015, Chen et al. 2019, Giese et al. 2020, Helf et al. 2022).
Data exclusions	No data were excluded.
Replication	- For comparative analysis of WT FAT-7::GFP and acdh-11(n5878);FAT-7::GFP mutants, more than five independent biological experiments were conducted, for which a subset were chosen as representative. In the representative experiment, in which N2 and fcmt-1(gk155709) were also grown in parallel, mixed-stage cultures of each genotype were split to three flasks each, and grown at 15 C, 20 C, or 25 C, resulting in the analysis of 4 exo-metabolome and 3 endo-metabolome samples for each genotype.<br>- For comparative analysis of acdh-11(n5878);FAT-7::GFP animals grown on BW25113 (WT) or JW1653-1 (cyclopropane-deficient) bacteria, four independent biological experiments were conducted, two of which included WT FAT-7::GFP grown in parallel. Additionally, supplementation with lactobacillic or dihydrosterculic acid was performed in parallel in two of the four experiments. Four additional independent biological experiments were conducted as part of the revision, in which acdh-11(n5878);FAT-7::GFP and WT FAT-7::GFP animals were grown in parallel and reared on BW25113 (WT) or JW1653-1 (cyclopropane-deficient) bacteria.<br>- For comparative analysis of N2 (laboratory strain) and the two fcmt-1 mutants, F13D12.9(gk155709) and F13D12.9(tm2382), synchronized adult animals from three independent biological experiments were analyzed, in which two technical replicates for each genotype were grown in parallel, with technical replicates extracted in two independent groups. One experiment contained only one replicate for the mutant F13D12.9(gk155709), resulting in 5 samples for this genotype. F13D12.9(gk155709) and N2 were additionally grown at different temperatures in parallel with WT FAT-7::GFP and acdh-11(n5878);FAT-7::GFP, as described above.<br>- For stable isotope labeling experiments, two independent experiments were conducted in which hacl-1(tm6725) larvae were supplemented with L-methionine or D3-methyl-L-methionine. One experiment was conducted in which a mixed-stage culture of hacl-1(tm6725) was split to seven cultures supplemented with vehicle, L-methionine, D3-methyl-L-methionine, cis-vaccenic acid, D13-cis-vaccenic acid, trans-vaccenic acid, or D13-trans-vaccenic acid. One additional experiment was conducted in which mixed-stage cultures of hacl-1(tm6725) and N2 (laboratory strain) were split to five cultures each and supplemented with vehicle, cis-vaccenic acid, D13-cis-vaccenic acid, trans-vaccenic acid, or D13-trans-vaccenic acid, for a total of three independent experiments for each isotope-labeled supplement.<br>- Microscopy was performed over 2+ years in more than 30 independent biological experiments across a range of diet, temperature,

genotype, and supplement conditions. After optimizing conditions, four independent biological experiments were quantified in which WT FAT-7::GFP was grown in parallel at 25 C under mock and 50 uM supplementation conditions. A representative FAT-6::GFP microscopy experiment is shown, from three biologically independent experiments.
- Four independent biological experiments were performed for RT-PCR analysis.
- Experiments were conducted over the course of 3+ years. All attempts at reproduction were successful.

Randomization	Samples were allocated into experimental groups based on genotype, supplement, and/or diet. In the majority of experiments, mutant and parental strains were grown in parallel (i.e., N2 and fcmt-1 mutants; FAT-7::GFP and FAT-7::GFP;acdh-11(n5878)). In any experiment investigating supplement or modified diet, animals were grown in parallel under reference conditions.
Blinding	Blinding was not possible during data collection because the acquired experimental data required manipulations of live animals. However, metabolomics data analysis was fully automated. For the quantification of fluorescence in microscopy experiments, animals were selected and outlined on the transmitted light micrographs, and then these outlines were transferred to GFP fluorescence micrographs, therefore there was no pre-selection based on a given animal's fluorescence intensity.

Reporting for specific materials, systems and methods

We require information from authors about some types of materials, experimental systems and methods used in many studies. Here, indicate whether each material, system or method listed is relevant to your study. If you are not sure if a list item applies to your research, read the appropriate section before selecting a response.

Materials & experimental systems

n/a	Involved in the study
☒	☐ Antibodies
☒	☐ Eukaryotic cell lines
☒	☐ Palaeontology and archaeology
☐	☒ Animals and other organisms
☒	☐ Clinical data
☒	☐ Dual use research of concern
☒	☐ Plants

Methods

n/a	Involved in the study
☒	☐ ChIP-seq
☒	☐ Flow cytometry
☒	☐ MRI-based neuroimaging

Animals and other research organisms

Policy information about studies involving animals; ARRIVE guidelines recommended for reporting animal research, and Sex and Gender in Research

Laboratory animals	The C. elegans laboratory strain N2 Bristol (wildtype); C. elegans mutant hacl-1(tm6725), strain designation FCS7; C. elegans mutant fcmt-1(gk155709), strain designation FCS40; C. elegans mutant fcmt-1(tm2382), strain designation FCS20; C. elegans nIs590 [Pfat-7::fat-7::GFP], strain designation DMS303; C. elegans acdh-11(n5878);nIs590, strain designation DMS441; C. elegans hacl-1 (tm6725);nIs590, strain designation FCS66; C. elegans lin-15B&lin-15A(n765) X; waEx16[Pfat-6::fat-6::GFP + 357 lin15(+)], strain designation BX115; and C. briggsae strain AF16. The age of the animals at harvest was typically between 1-3 days, with some older animals present in mixed-stage cultures.
Wild animals	No wild animals were used in this study.
Reporting on sex	No sex-based analyses were performed, C. elegans are predominantly self-fertilizing hermaphrodites grown for between 1-3 days prior to harvest.
Field-collected samples	No field-collected samples were used in this study.
Ethics oversight	No ethical approval was required.

Note that full information on the approval of the study protocol must also be provided in the manuscript.
